# Discovery of the β-barrel–type RNA methyltransferase responsible for *N*^6^-methylation of *N*^6^-threonylcarbamoyladenosine in tRNAs

**DOI:** 10.1093/nar/gku618

**Published:** 2014-07-24

**Authors:** Satoshi Kimura, Kenjyo Miyauchi, Yoshiho Ikeuchi, Patrick C. Thiaville, Valérie de Crécy-Lagard, Tsutomu Suzuki

**Affiliations:** 1 Department of Chemistry and Biotechnology, Graduate School of Engineering, University of Tokyo, 7-3-1 Hongo, Bunkyo-ku, Tokyo 113–8656, Japan; 2 Genetics and Genomics Graduate Program; 3 University of Florida Genetics Institute; 4 Department of Microbiology, University of Florida, Gainesville, Florida 32611–0700, USA; 5 Institut de Génétique et Microbiologie, Université of Paris-Sud, Orsay, France

## Abstract

Methylation is a versatile reaction involved in the synthesis and modification of biologically active molecules, including RNAs. *N*^6^-methyl-threonylcarbamoyl adenosine (m^6^t^6^A) is a post-transcriptional modification found at position 37 of tRNAs from bacteria, insect, plants, and mammals. Here, we report that in *Escherichia coli*, *yaeB* (renamed as *trmO*) encodes a tRNA methyltransferase responsible for the *N*^6^-methyl group of m^6^t^6^A in tRNA^Thr^ specific for ACY codons. TrmO has a unique single-sheeted β-barrel structure and does not belong to any known classes of methyltransferases. Recombinant TrmO employs S-adenosyl-*L*-methionine (AdoMet) as a methyl donor to methylate t^6^A to form m^6^t^6^A in tRNA^Thr^. Therefore, TrmO/YaeB represents a novel category of AdoMet-dependent methyltransferase (Class VIII). In a Δ*trmO* strain, m^6^t^6^A was converted to cyclic t^6^A (ct^6^A), suggesting that t^6^A is a common precursor for both m^6^t^6^A and ct^6^A. Furthermore, *N*^6^-methylation of t^6^A enhanced the attenuation activity of the *thr* operon, suggesting that TrmO ensures efficient decoding of ACY. We also identified a human homolog, TRMO, indicating that m^6^t^6^A plays a general role in fine-tuning of decoding in organisms from bacteria to mammals.

## INTRODUCTION

Methylation is a versatile and ubiquitous reaction involved in the synthesis and modifications of biological molecules including DNA, RNA, proteins, and other compounds. Methylation of DNA and histones is an essential aspect of epigenetic regulation of gene expression (1). Methylation of RNA and proteins plays modulatory roles in the biochemical and biophysical properties of these molecules (2,3). In addition, methylation is also involved in synthesis and/or conversion of various cellular metabolites, including some toxic compounds (4,5).

Methylation of biomolecules is catalyzed by methyltransferase (or methylase). Several families of methyltransferases exist, and they employ a variety of methyl-group donors, including methylcobalamin, methyl- and methylene-tetrahydrofolate, and S-adenosyl-*L*-methionine (AdoMet) (6). AdoMet-dependent methyltransferases, the largest family, are categorized into seven classes according to their structural motifs (6–8). Methyltransferases in this family frequently bear AdoMet-binding motifs, which have widely divergent amino acid sequences (9), suggesting that novel AdoMet-methyltransferases still remain to be discovered.

tRNAs contain numerous post-transcriptional modifications. The decoding abilities of tRNAs are modulated by modified bases in the anticodon region (10). The first letter of the anticodon (position 34) is subject to various chemical modifications, known as “wobble modifications,” which play an important role in regulating decoding capability. In addition, bulky modifications are frequently introduced at position 37, which is 3′-adjacent to the anticodon. The modified bases at position 37 stabilize codon–anticodon pairing via base-stacking interactions in the decoding center of the ribosome (11,12).

*N* ^6^-threonylcarbamoyladenosine (t^6^A) (Supplementary Figure S1) and its derivatives are universal modified bases present at position 37 of tRNAs responsible for codons starting with A (ANN codons) from all domains of life (13). The bulky structure of t^6^A supports formation of the canonical U-turn structure of the anticodon loop (14) by preventing U33-A37 base pairing (15). t^6^A plays a crucial role in maintaining decoding accuracy during protein synthesis, and it is also required for aminoacylation of tRNAs (16), tRNA binding to the A-site codon (17), efficient translocation (18), reading-frame maintenance (19), and prevention of leaky scanning of initiation codons and read-through of stop codons (20). Biogenesis of t^6^A has been extensively studied. In bacterial systems, the formation of t^6^A on tRNA was successfully reconstituted *in vitro* using four essential enzymes, TsaC (YrdC), TsaD (YgjD), TsaB (YeaZ), and TsaE (YjeE), in the presence of the substrates *L*-threonine, ATP, and bicarbonate (21). In the first step of this reaction, TsaC employs *L*-threonine, bicarbonate, and ATP to synthesize threonylcarbamoyl-adenylate (TC-AMP), an active intermediate in t^6^A formation (22). Next, the three other enzymes (TsaD, TsaB, and TsaE) catalyze nucleophilic attack of the *N*^6^-amino group of A37 on the carbonyl group of TC-AMP to synthesize t^6^A, releasing AMP as a leaving group. In eukaryotes and archaea, the TsaC/YrdC homolog Sua5 and several components of the KEOPS/EKC complex, including Kae1, Pcc1, and Bud32, are involved in formation of t^6^A (19,23–28). On the other hand, in mitochondria, the TsaD/YgjD homolog Qri7 is the sole enzyme responsible for the second step of t^6^A formation (29,30).

**Figure 1.**
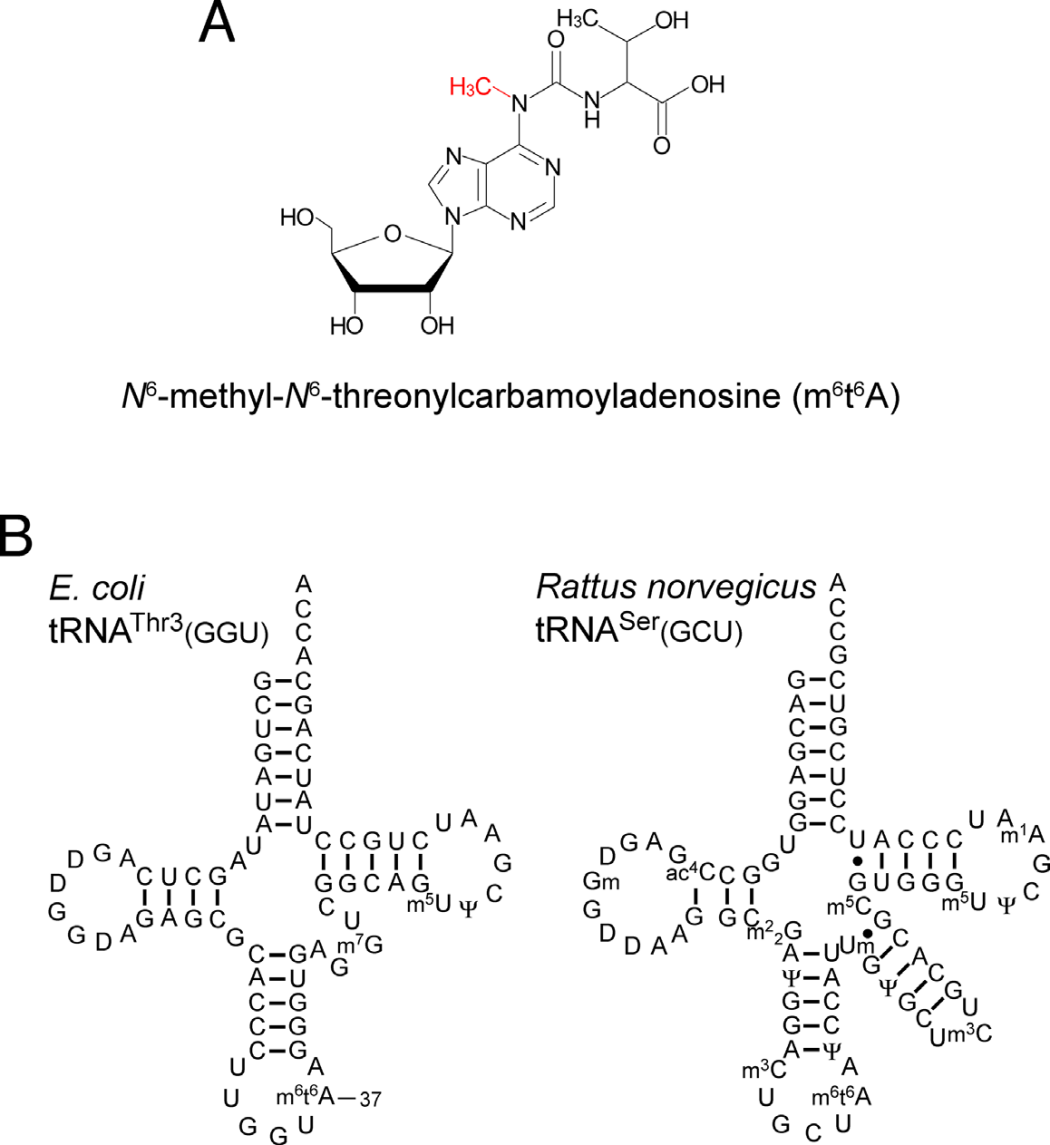
*N*^6^-methyl-*N*^6^-threonylcarbamoyl adenosine (m^6^t^6^A) and tRNAs bearing m^6^t^6^A. (A) Chemical structure of m^6^t^6^A. The *N*^6^-methyl group is shown in red. (B) Secondary structures of *E. coli* tRNA^Thr3^(GGU) and *Rattus norvegicus* tRNA^Ser^ (GCU) with post-transcriptional modifications: dihydrouridine (D), *N*^6^-methyl-*N*^6^-threonylcarbamoyladenosine (m^6^t^6^A), 7-methylguanosine (m^7^G), 5-methyluridine (m^5^U), pseudouridine (Ψ), *N*^4^-acetylcytidine (ac^4^C), 2′-*O*-methylguanosine (Gm), *N*^2^, *N*^2^-dimethylguanosine (m^2^_2_G), 3-methylcytidine (m^3^C), 2′-*O*-methyluridine (Um), 5-methylcytidine (m^5^C), and 1-methyladenosine (m^1^A).

Although the presence of t^6^A in cellular tRNAs from *E. coli* and yeast has been well documented for more than four decades, we recently showed that t^6^A is a hydrolyzed artifact of cyclic t^6^A (ct^6^A) (Supplementary Figure S1), a bona fide modified base of *E. coli* tRNAs (31). ct^6^A is widely distributed among tRNAs from a certain group of bacteria, fungi, plants, and some protists, whereas t^6^A is present in tRNAs of mammals, archaea, and other group of bacteria. In *E. coli* cells, almost all t^6^A is converted to ct^6^A via a dehydration reaction catalyzed by TcdA. Thus, ct^6^A is an additional modification of t^6^A that enhances tRNA-decoding activity. In addition to ct^6^A, two other derivatives of t^6^A exist: 2-methylthio-*N*^6^-threonylcarbamoyladenosine (ms^2^t^6^A) (Supplementary Figure S1), found in tRNA^Lys^ from *Bacillus subtilis* and mammals (32,33), and *N*^6^-methyl-*N*^6^-threonylcarbamoyladenosine (m^6^t^6^A) (Figure 1A), found in tRNAs from bacteria, fly, plants, and rat (Figure 1B) (34,35). The methylthiolation of ms^2^t^6^A is required for the accurate decoding of lysine codons. *B. subtilis* MtaB (36,37) and human Cdkal1 (38) serve as the methylthiotransferases responsible for introducing ms^2^t^6^A into tRNA. In addition, variation in Cdkal1 is associated with risk of type 2 diabetes (38). In *E. coli*, m^6^t^6^A is present at position 37 of tRNA^Thr1^(GGU) and tRNA^Thr3^(GGU), both of which decipher ACY codons (39,40) (Figure 1B and Supplementary Figure S2), whereas the isoacceptors tRNA^Thr2^(CGU) and tRNA^Thr4^(UGU) contain ct^6^A37 (31) (Supplementary Figure S2).

*N* ^6^-methylation of m^6^t^6^A plays a role in efficient attenuation of the *thrABC* operon (41). The expression level of *thrABC* is regulated by translation of a leader peptide encoded by *thrL*, which contains consecutive ACC codons. The mutant strain *tsaA*, which lacks *N*^6^-methylation of m^6^t^6^A37, exhibits relieved attenuation of *thrABC* (41), probably due to inefficient translation of the ACC codons in ThrL. The *tsaA* gene is predicted to be located at 4.6 min in the *E. coli* genome (41). Although the methyl donor responsible for *N*^6^-methylation of m^6^t^6^A37 was shown to be AdoMet (41), no AdoMet-methyltransferase was identified near 4.6 min in the *E. coli* genome during the 15 years following that discovery.

We have been screening for genes responsible for RNA modifications using ‘ribonucleome analysis’ (42), a reverse-genetics approach combined with mass spectrometry. The screen has identified a number of genes involved in tRNA modifications (43–48) and rRNA modifications (49,50). In this study, by performing ribonucleome analysis in *E. coli*, we demonstrate that *tsaA* is found to be *yaeB* which is responsible for *N*^6^-methylation of m^6^t^6^A in tRNA^Thr^. Using AdoMet as a methyl donor, recombinant YaeB methylated the *N*^6^ position of t^6^A to form m^6^t^6^A in tRNA^Thr^. Therefore, we renamed YaeB as TrmO (tRNA-methyltransferase O). TrmO, which contains a single-sheeted β-barrel structure and does not belong to any known class of AdoMet methyltransferases, is the founding member of a novel class (VIII) of AdoMet-dependent methyltransferases. We here characterize TrmO in terms of evolutionary distribution, tRNA-decoding ability and substrate specificity. In addition, we identified a human homolog TRMO.

## MATERIALS AND METHODS

### Strains and medium

*E*. *coli* genomic-deletion strains (OCL/R-series) derived from MG1655sp (MG1655 *rpsL polA12 Zih*::Tn10), each lacking about 20 kbp (∼20 genes), were kindly provided by Dr. Junichi Kato. OCR36 [MG1655 Δ(OCR36–1)::Kan/p36–4] specifically lacked m^6^t^6^A. A series of single-deletion strains with the kanamycin-resistance marker (Km^r^) were obtained from the Genetic Stock Research Center, National Institute of Genetics, Japan. The Δ*trmO*::Cm^r^ strain was constructed by one-step gene disruption (51) utilizing the chloramphenicol-resistance marker (Cm^r^) from pBT (Invitrogen). The Δ*trmO*/Δ*tcdA* double-deletion strain was constructed by P1 transduction of Δ*tcdA*::Km^r^ to the Δ*trmO*::Cm^r^ strain. The primers are listed in Supplementary Table S2. *E. coli* strains were grown in 5 ml of LB medium at 37°C overnight.

### RNA mass spectrometry

Total RNA was extracted from the cells by the AGPC method (52) using ISOGEN (Nippon Gene, Japan) or Tripure (Roche). Nucleoside analysis of the extracted RNAs was performed by LC/MS using an LCQ Advantage ion-trap mass spectrometer (Thermo Fisher Scientific) equipped with an ESI source and an HP1100 liquid chromatography system (Agilent Technologies), as described previously (42). RNA fragments of the isolated tRNAs digested by RNases were analyzed by capillary LC/nano ESI-MS as described (31,42,53). In brief, 1 pmol of isolated tRNA was digested with 50 units of RNase T_1_ (Epicentre) in 20 mM NH_4_OAc (pH 5.3) at 37 °C for 30 min. The digests were analyzed using an LTQ Orbitrap mass spectrometer (Thermo Scientific) with a nano-electrosprayer connected with a splitless nanoflow HPLC system (DiNa, KYA Technologies).

### Isolation of individual tRNAs

For each sample, the cell pellet was resuspended in 5 ml RNA extraction buffer [50 mM NaOAc (pH 5.2) and 10 mM Mg(OAc)_2_ (pH 5.2)], mixed with 5 ml water-saturated phenol, and vigorously stirred for 60 min. The aqueous phase was separated by centrifugation and washed with chloroform. RNA was extracted with an equal volume of Tripure (Roche) and about 0.2 volume of chloroform, and then precipitated with 2-propanol. The RNA pellet was dissolved in deionized water and precipitated again with ethanol. The resultant pellet was rinsed with 80% ethanol and dried. Individual tRNAs were isolated by reciprocal circulating chromatography (RCC) using an automatic RCC device, basically following the previously described method (54). The 5′-terminal ethylcarbamate amino-modified DNA probes, 5′-TGGTGCTGATACCCAGAGTCGAACTGGGGA-3′ for *E. coli* tRNA^Thr1^(GGU), 5′-TGGTGCTGATAGGCAGATTCGAACTGCCGA-3′ for *E. coli* tRNA^Thr3^(GGU), and 5′-TGGATTAGCAGTCCATCGCCTTAACCACTCGGCCA-3′ for human tRNA^Ser^(GCU) were covalently immobilized on NHS-activated Sepharose 4 Fast Flow (GE Healthcare). DNA resins were packed into the custom-made tips attached to a multichannel head on an RCC device.

### Nucleoside preparation

Total nucleosides containing ct^6^A were usually prepared by neutral one-step digestion of total RNA (31). Total RNA (40 μg) was digested at 37°C for 1.5 h in 20 mM HEPES-KOH (pH 7.1) containing 0.1 U Nuclease P1 (Wako Pure Chemical Industries, Ltd.) and 0.08 U bacterial alkaline phosphatase (BAP) (*E. coli* C75, Wako Pure Chemical Industries, Ltd.). For the analysis of the *trmO* mutation, total RNA was completely digested by three-step digestion, as follows. Total RNA (40 μg) was incubated at 37°C for 1 h in 25 mM NH_4_OAc (pH 5.3) containing 0.1 unit Nuclease P1. Thereafter, 0.1 volume of 1 M ammonium bicarbonate (pH 8.0) with 0.127 units of phosphodiesterase I (PDase I) (Worthington Biochemical Corporation) was added to the mixture, followed by incubation at 37°C for 1 h. Finally, 0.08 U BAP was added, and the sample was incubated at 37°C for 1.5 h. Prior to use, Nuclease P1, BAP and phosphodiesterase I were dialyzed against deionized water and stored at -30°C.

### Preparation of t^6^A-containing tRNA transcript

*E. coli* tRNA^Thr3^(GGU), tRNA^Thr4^(UGU) and their derivatives were prepared by *in vitro* transcription using T7 RNA polymerase (55). Recombinant T7 RNA polymerase with N-terminal His-tag was expressed as a soluble form in *E. coli*, and purified with nickel-cheleting affinity chromatography in our labolatory. DNA templates for *in vitro* transcription were constructed by PCR using synthetic oligo DNAs (Supplementary Table S2). *In vitro* transcription was performed at 37°C for 3–6 h in a reaction mixture containing 40 mM Tris-HCl (pH 8.0), 5 mM dithiothreitol, 2 mM spermidine, 24 mM MgCl_2_, 0.01% Triton X-100, 2 mM NTPs, 10 mM 5′-monophosphoguanosine (GMP), template DNA, and T7 RNA polymerase. Each transcript was purified by electrophoresis on a 10% polyacrylamide gel containing 7 M urea, and then eluted in buffer consisting of 300 mM sodium acetate (pH 5.2), 0.1% SDS, and 1 mM EDTA (pH 8.0).

*In vitro* reconstitution of t^6^A was carried out essentially as described (21,56). t^6^A37 was introduced to each tRNA transcript at 37°C for 3 h in a 200 μl reaction mixture consisting of 25 μg of tRNA transcript, 1.5 μM each of the t^6^A-modifying enzymes (YrdC, YgjD, YeaZ and YjeE), 1 mM *L*-threonine, 2 mM ATP, 25 mM NaHCO_3_, 100 mM HEPES-KOH (pH7.5), 300 mM KCl, 20 mM MgCl_2_, and 5 mM DTT. The modified tRNA was extracted with ISOGEN (Nippon Gene, Japan) or Tripure (Roche), and then dialyzed against deionized water. The degree of t^6^A in each tRNA was analyzed by LC/MS RNA fragment analysis, as described above.

### Expression and purification of recombinant proteins

An *E. coli* strain carrying plasmid pCA24N, for expression of soluble recombinant TrmO protein fused to a N-terminal 6×His tag, was obtained from the ASKA clone collection [NBRP (NIG, Japan): *E. coli*] (57). This strain was cultivated in the presence of 0.1 mM IPTG to induce protein expression, and the expressed protein was puriﬁed using Ni-NTA beads (QIAGEN) packed in an open column. The pooled protein was dialyzed against a buffer consisting of 50 mM Tris–HCl (pH 7.5), 1 mM DTT, and 100 mM KCl.

The cDNA encoding the human homolog TRMO, obtained by nested RT-PCR using specific primers (Supplementary Table S2) from total RNA of HeLa cells, was cloned into the *Nhe*I and *Sal*I sites of vector pET28a to yield pET28a-TRMO (Novagen). BL21 (DE3) Rosetta was transformed with pET28a-TRMO, and the transformant strain was cultured at 37°C. When OD_600_ reached 0.7, expression of the recombinant protein was induced with 0.1 mM IPTG at 18°C for 3 h. The harvested cells were lysed by sonication in a buffer containing 25 mM Tris-HCl (pH 8.0), 300 mM NaCl, 10% glycerol, and 0.2 mM phenylmethylsulfonyl fluoride (PMSF). Recombinant TRMO was purified with Ni-NTA beads (QIAGEN). TRMO was eluted with 250 mM imidazole, and then passed through a PD-10 column (GE Healthcare) in cell lysis buffer containing 1 mM DTT.

### Gel retardation assay

The gel retardation assay was performed essentially as described (43,45). Recombinant TrmO (15–45 pmol) and *in vitro* transcribed tRNA (15 pmol) were incubated at 37°C for 30 min in a 10 μl mixture containing 50 mM Tris-HCl (pH 8.0), 5 mM MgCl_2_, 100 mM KCl, and 2 mM spermine. The tRNA–protein complex was resolved by 4% native polyacrylamide gel electrophoresis with a running buffer consisting of 50 mM Tris and 5 mM Mg(OAc)_2_ (pH 8.0, adjusted with acetic acid). After electrophoresis, the gel was stained with SYBR gold (Invitrogen) to visualize tRNA, and then stained with Coomassie Brilliant Blue (Nacalai Tesque) to visualize the protein.

### *In vitro N* ^6^-methylation of t^6^A by TrmO

For *in vitro* methylation (as shown in Figure 3B–D), 1 μM tRNA^Thr3^(GGU) with or without t^6^A was incubated at 37°C for 1 h in a 20 μl reaction mixture containing 50 mM HEPES-KOH (pH 6.7), 100 mM KCl, 5 mM MgCl_2_, 1 mM DTT, 10 μM TrmO, and 1 mM AdoMet. Modified tRNA extracted with Tripure was digested with RNase and analyzed by LC/MS as described above. For methylation by TRMO (Figure 6E and F), the same conditions were used, except that the reaction volume was 100 μl and the enzyme was 1 μM TRMO. For a tRNA mutation study (Figure 4), 1 μM tRNAs bearing t^6^A37 were incubated at 37°C for 5 min in a 10 μl reaction mixture containing 50 mM HEPES-KOH (pH 6.7), 100 mM KCl, 5 mM MgCl_2_, 1 mM DTT, 0.1 μM TrmO, and 25 μM [^14^C-methyl]AdoMet (1.74 Gbq/mmol, Perkin Elmer). The modified tRNAs were extracted with phenol–chloroform–isoamyl alcohol (Nacalai Tesque) and spotted on filter paper (Whatman 3MM). The filter papers were washed three times with 5% trichloroacetic acid, and then soaked with 100% ethanol. After drying, radioactivity on the filter papers was measured by liquid scintillation counting.

**Figure 2.**
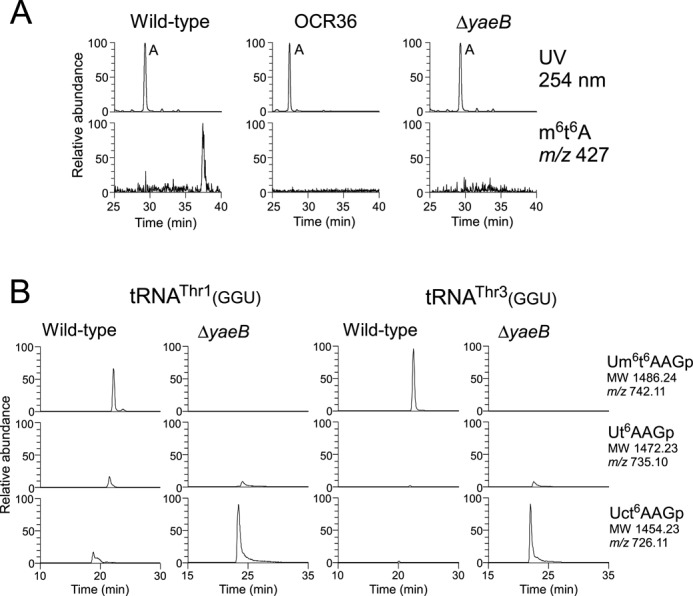
*E. coli yaeB* is responsible for m^6^t^6^A formation. (A) *yaeB* is the gene altered in the *tsaA* mutant. LC/MS analysis of total nucleosides extracted from wild type (left panels), OCR36 (middle panels), and Δ*yaeB* (right panels).Upper and lower panels show UV traces at 254 nm and mass chromatograms for the proton adduct of m^6^t^6^A (*m/z* 427). (B) Mass chromatograms of the RNA fragments containing position 37 of tRNA^Thr1^(GGU) (left panels) and tRNA^Thr3^(GGU) (right panels) isolated from *E. coli* wild type and Δ*yaeB*. Top, middle, and bottom panels show doubly charged negative ions of m^6^t^6^A-containing tetramer (Um^6^t^6^AAGp; MW 1486.24, *m*/*z* 742.11), t^6^A-containing tetramer (Ut^6^AAGp; MW 1472.23, *m*/*z* 735.10), and ct^6^A-containing tetramer (Uct^6^AAGp; MW 1454.23, *m*/*z* 726.11), respectively.

**Figure 3.**
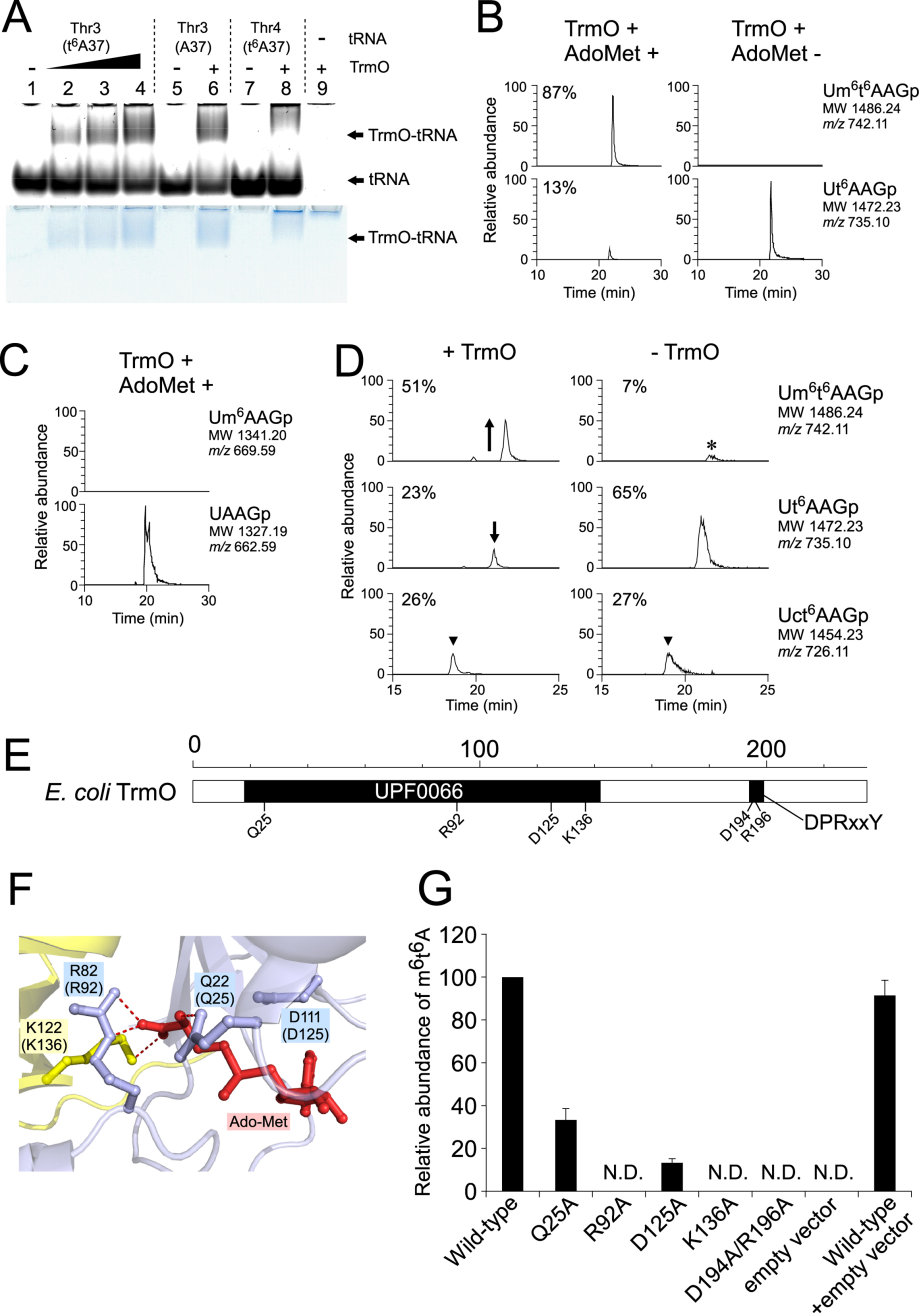
TrmO is an AdoMet-dependent methyltransferase responsible for m^6^t^6^A formation. (A) Physical interaction of TrmO and tRNAs by gel-retardation assay. The polyacrylamide gel was stained with SYBR gold (upper panel) and Coomassie brilliant blue (lower panel). Lanes 1–4 represent tRNA^Thr3^(GGU) (t^6^A37) with 0, 15, 30, and 45 pmol TrmO; lanes 5 and 6 represent tRNA^Thr3^(GGU) (A37) without or with TrmO (45 pmol). Lanes 7 and 8 represent tRNA^Thr4^(UGU) (t^6^A37) without or with TrmO (45 pmol). Lane 9 represents TrmO (45 pmol) only. All conditions contained 15 pmol tRNA. (B) *In vitro* methylation by TrmO. *E. coli* tRNA^Thr3^(GGU) transcript bearing t^6^A37 was incubated in the presence of recombinant TrmO with (left panels) or without (right panels) AdoMet. Upper and lower panels: mass chromatograms showing doubly-charged negative ions of m^6^t^6^A-containing tetramer (Um^6^t^6^AAGp; MW 1486.24, *m*/*z* 742.11) and t^6^A-containing tetramer (Ut^6^AAGp; MW 1472.23, *m*/*z* 735.10), respectively. (C) The same experiment as in B, using *E. coli* tRNA^Thr3^(GGU) transcript bearing A37 (without t^6^A). No methylation took place in this tRNA, even in the presence of both TrmO and AdoMet. (D) The same experiment as in B, using *E*. *coli* tRNA^Thr3^(GGU) isolated from the Δ*trmO* strain. The peak marked with an asterisk represents the m^6^t^6^A-containing fragment derived from carryover of wild-type *E*. *coli* tRNA^Thr3^(GGU) bound to the oligo DNA probe. (E) Schematic domain structure of *E. coli* TrmO. Scale denotes amino acid numbering. (F) Close-up view of the AdoMet-binding site in the crystal structure of *A. fulgidus* AF0241. Bound AdoMet is shown as a red stick. Four amino acid residues that interact with AdoMet are colored. Note that only K122 (yellow) is a residue from the other subunit. The amino acid numbers of *E. coli* TrmO are shown in parentheses. Possible hydrogen bonds are shown as dash lines. (G) Mutation study of *trmO*. The Δ*trmO* strain was transformed with plasmid-encoded *trmO* wild type or mutants. The wild-type strain (BW25113) was also transformed with the empty vector (pHSG415r). The height of the mass chromatogram for the proton adduct of m^6^t^6^A (*m/z* 427) in each transformant was divided by that of m^2^A (*m/z* 282), and the relative ratio was normalized to the result of Δ*trmO* complemented with wild-type *trmO*. The bar graph show the average value of three experiments, and error bars indicate the SD values. N.D., not detected.

**Figure 4.**
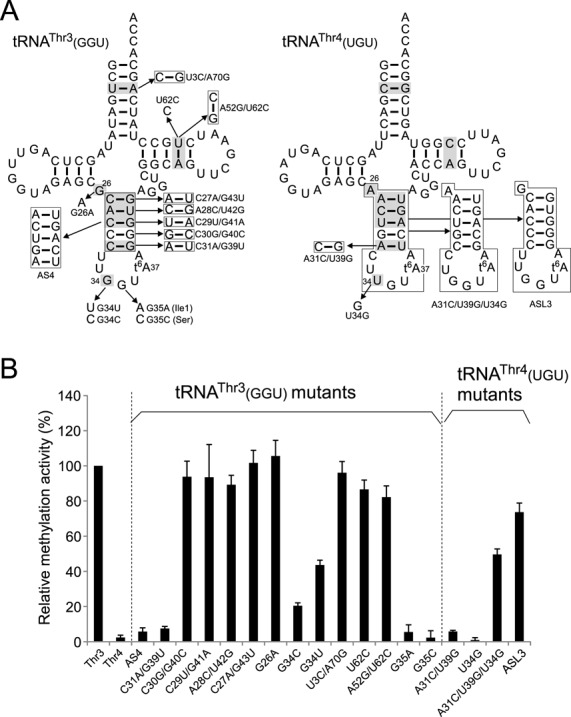
Mutation study to elucidate the mechanism of tRNA discrimination by TrmO. (A) Variants of *E. coli* tRNA^Thr3^(GGU) (left-hand side) and tRNA^Thr4^(UGU) (right-hand side) used in this study. The numbering system of tRNA is based on the tRNA compilation (79). Bases common to tRNA^Thr1,3^ but different in tRNA^Thr2,4^ are highlighted in gray in each tRNA. (B) Relative methylation activities of TrmO for tRNA variants normalized by the activity of wide-type tRNA^Thr3^(GGU) (Thr3). Radioactivity of the [^14^C] methyl group incorporated into each tRNA variant over the course of a 5 min reaction was measured. The averaged values of three independent experiments, with SD values, are shown.

**Figure 5.**
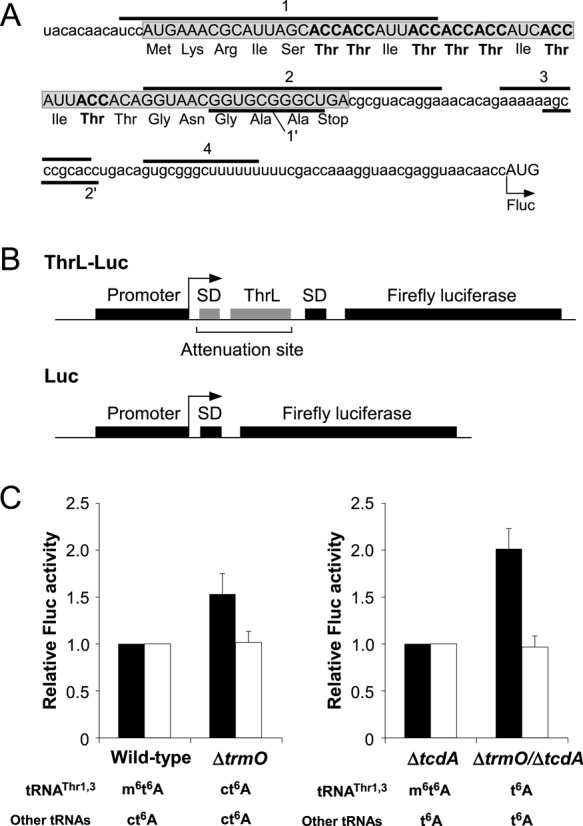
*N*^6^-methylation of t^6^A enhances the decoding ability of tRNA^Thr^. (A) mRNA sequence of the attenuation site in the *thr* operon. The amino acid sequence denotes the ThrL leader peptide (boxed and shaded ORF). ACC codons and Thr residues are shown in bold. Segments 1 and 2 potentially form hairpin-like structures. Segments 3 and 4 form a stable terminator hairpin that halts transcription. Segments 1′ and 2′ also form a stable duplex that acts as an anti-terminator by preventing the formation of the terminator hairpin mediated by segments 3 and 4. When the ThrL leader peptide is actively translated, the terminator hairpin formed by segments 3 and 4 is stabilized, and the transcription of the downstream gene (in this case, firefly luciferase) is attenuated. On the other hand, when the consecutive ACC codons in the ThrL leader are not translated efficiently, the stable duplex formed by segments 1′ and 2′ prevents terminator hairpin formation, leading to efficient transcription of the downstream gene. (B) Schematic depiction of the reporter constructs for ThrL-Luc and Luc. (C) Relative attenuation activity of the ThrL-Luc reporter in Δ*trmO*. Relative firefly luciferase (Fluc) activity of ThrL-Luc (black bars) or Luc (white bars) reporter was normalized to the OD_600_ of the culture. Relative Fluc activities of Δ*trmO* and Δ*trmO/*Δ*tcdA* were normalized to those of wild-type and Δ*tcdA*, respectively. The averaged values of four independent experiments, with SD values, are shown. The expected modification status at position 37 of tRNA^Thr1,3^ and other tRNAs is indicated under the data for each strain.

**Figure 6.**
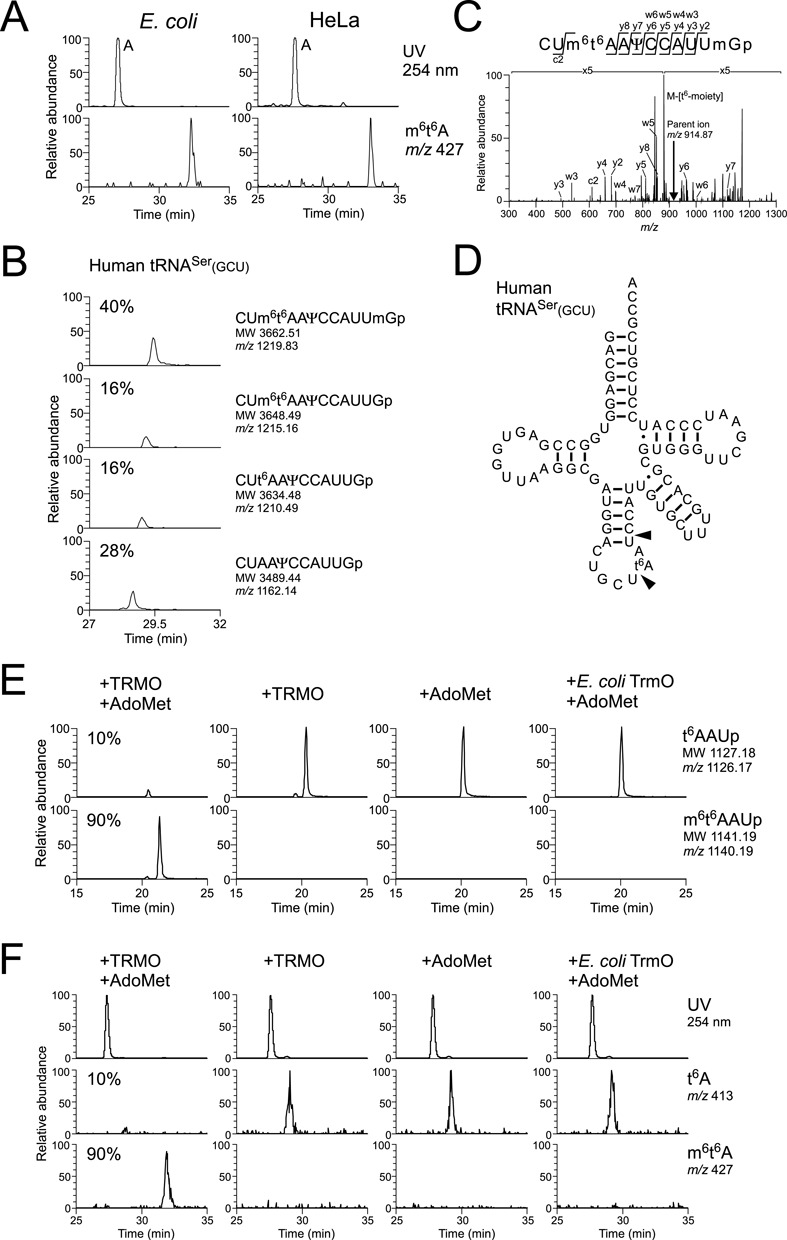
Human homolog TRMO catalyzes m^6^t^6^A formation in tRNA^Ser^(GCU). (A) LC/MS nucleoside analysis of wild-type *E. coli* (left panels) and HeLa cells (right panels). Upper panels show UV traces at 254 nm, and lower panels show mass chromatograms for the proton adduct of m^6^t^6^A (*m/z* 427). (B) LC/MS analysis of RNase T_1_ digested human cytoplasmic tRNA^Ser^(GCU). All panels are mass chromatograms for triply-charged negative ions. From upper to bottom panels, m^6^t^6^A37- and Um43-containing fragment (CUm^6^t^6^AAΨCCAUUmGp; MW 3662.51, *m/z* 1219.83), m^6^t^6^A-containing fragment (CUm^6^t^6^AAΨCCAUUGp; MW 3648.49, *m/z* 1215.16), t^6^A-containing fragment (CUt^6^AAΨCCAUUGp; MW 3634.48, *m/z* 1210.49) and unmodified fragment (CUAAΨCCAUUGp; MW 3489.44, *m/z* 1162.14). Ψ39 and Um44 are predicted from the sequence of rat tRNA^Ser^(GCU). (C) CID spectrum of m^6^t^6^A- and Um-containing fragment of human cytoplasmic tRNA^Ser^(GCU). (D) Transcript of human cytoplasmic tRNA^Ser^(GCU) bearing t^6^A37 used for *in vitro* reconstitution of m^6^t^6^A37. Arrowheads indicate cleavage sites by RNase A. (E) *In vitro* methylation by recombinant TRMO. Human cytoplasmic tRNA^Ser^(GCU) transcript bearing t^6^A37 was incubated with TRMO and AdoMet (left most panels), TRMO only (left middle panels), AdoMet only (right middle panels) or *E. coli* TrmO and AdoMet (right most panels). Upper and lower panels show mass chromatograms for singly charged negative ions of t^6^A-containing trimer (t^6^AAUp; MW 1127.18, *m*/*z* 1126.17) and m^6^t^6^A-containing trimer (m^6^t^6^AAUp; MW 1141.19, *m*/*z* 1140.19), respectively. (F) LC/MS nucleoside analysis of human cytoplasmic tRNA^Ser^(GCU) transcript bearing t^6^A37, which was incubated with TRMO and AdoMet (left most panels), TRMO only (left middle panels), AdoMet only (right middle panels), or *E. coli* TrmO and AdoMet (right most panels). Upper panels show UV trace at 254 nm. Middle and lower panels show mass chromatograms for the proton adduct of t^6^A (*m/z* 413) and m^6^t^6^A (*m/z* 427), respectively.

### Plasmid complementation

The *E. coli trmO* gene with 5′ and 3′ flanking regions, probably including its original promoter and terminator, was amplified by PCR using specific primers (Supplementary Table S2). The PCR product was cloned into the *Eco*RI and *Xho*I sites of pHSG415r, which contains a pSC101 origin, to yield pHSG-TrmO. Mutations were introduced into pHSG-TrmO by site-directed mutagenesis using PrimeSTAR HS DNA polymerase (TaKaRa) using specific primers (Supplementary Table S2). Each mutant was checked by Sanger sequencing. The Δ*trmO* strain was transformed with each plasmid, and the resultant transformants were cultivated to mid-log phase. Total RNA from each construct was digested to nucleosides and analyzed by LC/MS, as described above. The height of the mass chromatogram for the proton adduct of m^6^t^6^A (*m/z* 427) was divided by the height of the mass chromatogram for m^2^A (*m/z* 282), and the relative ratio was normalized to the result from the Δ*trmO* complemented with wild-type pHSG-TrmO.

### Luciferase assay

The luciferase assay was performed as described previously (50). A reporter containing the *thrL* attenuator followed by firefly luciferase gene (ThrL-Luc), and a control reporter without the attenuator (Luc), were constructed as follows. The attenuation site of the *thr* operon including the promoter, leader peptide (*thrL*), and terminator was amplified by PCR using specific primers (Supplementary Table S2). The ORF of firefly luciferase was also amplified using specific primers (Supplementary Table S2). These PCR products and linearized pBR322 digested with *Eco*RI and *Bam*HI were ligated using an In-Fusion HD Cloning Kit (TaKaRa). The control reporter without the attenuator (Luc) was generated by deleting the attenuation site using specific primers (Supplementary Table S2) and the PrimeSTAR HS DNA polymerase (TaKaRa). The sequences of the resultant plasmids were verified by Sanger sequencing.

*E*. *coli* wild-type (BW25113), Δ*trmO*, Δ*tcdA* and Δ*trmO*/Δ*tcdA* strains were transformed with each of the reporters. Each transformant was cultivated in 2 ml LB liquid medium at 37°C. When the cultures reached 0.4–0.6 OD_600_, 0.5 ml of the culture was harvested and resuspended in 100 μl lysis buffer [50 mM HEPES-KOH (pH 7.6), 100 mM KCl, 10 mM MgCl_2_, 7 mM β-mercaptoethanol, and 400 μg/ml lysozyme]. Cell lysates were prepared by the freeze-thaw method and cleared by centrifugation (15 min; 15,000 rpm, 4°C). Cleared lysates (5 μl) were analyzed with a GLOMAX96 Microplate Luminometer (Promega) using the Dual-Luciferase Reporter Assay System (Promega). The efficiency of attenuation was measured by the chemiluminescence of firefly luciferase, normalized to the OD_600_ of the culture.

### Phylogenetic analysis and taxonomic distribution of *yaeB*

The number of *yaeB* homologs in each of the 955 taxa present in the SEED database (58) were retrieved from the “COG1720” subsystem. All taxa with a *yaeB* homolog are listed in Supplementary Table S1. Taxonomic distribution of *yaeB* homologs, pruned to the level of orders, is shown in Supplementary Figure S4. The data were formatted to contain only NCBI taxonomic identification numbers and the number of occurrences of *yaeB* in each taxon. The amino acid sequences of all COG1720 proteins were downloaded from SEED and converted to the nexus format using the University of Florida's High Performance Cluster (UFHPC) instance of Galaxy (galaxy.hpc.ufl.edu) (59–61). Alignments were trimmed and refined using Se-Al (tree.bio.ed.ac.uk/software/seal). The 19 eukaryotic members of COG1720 did not produce quality alignments and were excluded from further analysis. Additionally, proteins from 11 archaea and 70 bacteria were truncated or were missing conserved residues (Gly7, Pro74, Asn75, Asp107 for Archaea, and Gln17, Pro89, Asn90, Asp121 for Bacteria), and were also excluded from further analysis. Alignments used in phylogenetic analysis are included in supplementary material and include 216 bacterial and 60 archaeal taxa. Phylogenetic analysis was performed using MrBayes 3.2.1 (62) on the University of Florida High Performance Cluster (hpc.ufl.edu) using the Dayhoff-6 amino acid categories and inferred a tree with the CAT+Γ model to account for evolutionary rate site variations. MrBayes was run for 1 000 000 or 2 000 000 (for combined bacterial and archaeal tree) MCMC iterations with a 10% burnin. Trees were sampled every 1000 iterations, and a consensus tree was generated by MrBayes using 50% majority rule. Convergences of the runs were checked with Tracer (tree.bio.ed.ac.uk/software/tracer). Consensus trees were visualized using iTOL (itol.embl.de) with posterior probabilities displayed on the branches.

## RESULTS

### Identification of *yaeB* gene responsible for *N*^6^-methylation of t^6^A37

To identify genes responsible for tRNA/rRNA modifications, we have performed screens employing a combination of reverse genetics and mass spectrometry, a strategy we call ‘ribonucleome analysis’ (42). In this type of screen, we use LC/MS to analyze modified nucleosides in total RNA from a series of genome-deletion strains. In the initial screen, which analyzed 130 genomic-deletion strains covering approximately 50% of *E*. *coli* open reading frames (ORFs), we demonstrated that m^6^t^6^A was specifically absent in a particular genomic-deletion strain, OCR36, which covers 4.72–5.25 min of the *E*. *coli* genome (Figure 2A). The deleted region of OCR36 encodes 23 ORFs, including ten uncharacterized genes, three rRNA genes, and four tRNA genes. However, this region did not contain any known class of AdoMet-dependent methyltransferases. We analyzed strains bearing single-gene deletions in each of ten uncharacterized genes and found that m^6^t^6^A was specifically absent in the Δ*yaeB* strain (Figure 2A), suggesting that *yaeB* is involved in m^6^t^6^A formation. Based on this finding, *yaeB* was found to be the gene altered in the *tsaA* mutant (41). In parallel, we identified *yaeB* in the *tsaA* mutant by genetic mapping (Supplementary result).

Next, we isolated tRNA^Thr1^(GGU) and tRNA^Thr3^(GGU) from the wild-type and Δ*yaeB* strains, and analyzed the RNase T_1_-digested fragments by LC/MS. The m^6^t^6^A-containing tetramer (Um^6^t^6^AAGp) was clearly present in both tRNAs isolated from the wild-type strain (Figure 2B). In tRNAs from Δ*yaeB*, however, no m^6^t^6^A-containing tetramer was detected; instead, we observed a ct^6^A-containing tetramer (Uct^6^AAGp), as well as a small fraction of t^6^A-containing tetramer (Ut^6^AAGp) (Figure 2B). The results indicate that in the absence of *yaeB*, tRNA^Thr1^(GGU) and tRNA^Thr3^(GGU) can serve as substrates for TcdA, which catalyzes dehydration of t^6^A to form ct^6^A(31). Therefore, t^6^A is a common precursor for m^6^t^6^A and ct^6^A.

### TrmO is an AdoMet-dependent methyltransferase responsible for m^6^t^6^A formation.

According to the NCBI protein database, *yaeB* contains the uncharacterized protein domain UPF0066. Homologs of *yaeB* are widely distributed in bacteria, archaea, and eukaryotes. Crystal structures of three homologs, AF0241 (*Archaeoglobus fulgidus*)(63), RPA0152 (*Rhodopseudomonas palustris*), and HI0510 (*Haemophilus influenzae*), have been solved and deposited in PDB (Supplementary Figure S3). All of these homologs have single-sheeted, anti-parallel β-barrel structures and form homodimers. Intriguingly, two of these structures (of homologs AF0241 and RPA0152) contained endogenous AdoMet molecules, strongly suggesting that YaeB is an AdoMet-dependent methyltransferase.

To determine whether YaeB is the tRNA methyltransferase physiologically responsible for *N*^6^-methylation of m^6^t^6^A, we recombinantly expressed YaeB and purified it to homogeneity. To generate substrates, we prepared tRNA transcripts by *in vitro* transcription, and introduced t^6^A at position 37 by *in vitro* enzymatic reaction (21,56) (see Materials and Methods). At the outset, we examined the ability of YaeB to interact with tRNAs in gel-mobility shift experiments. A tRNA–protein complex was clearly observed for tRNA^Thr3^(GGU) containing t^6^A37, and a larger amount of this complex was formed in the presence of higher levels of recombinant YaeB (Figure 3A), demonstrating that YaeB directly recognizes tRNA^Thr3^(GGU). Because the efficiency of complex formation was not altered when unmodified tRNA^Thr3^(GGU) was used for this experiment, we concluded that YaeB does not require t^6^A37 to interact with tRNA^Thr3^(GGU). When tRNA^Thr4^(UGU) containing t^6^A37 was mixed with recombinant YaeB, the tRNA–protein complex appeared to be unstable, yielding a smeared band. These results indicate that YaeB preferentially binds to tRNA^Thr3^(GGU).

Next, we performed *in vitro* reconstitution of m^6^t^6^A. The tRNA^Thr3^(GGU) bearing t^6^A37 was incubated with recombinant YaeB in the presence or absence of AdoMet. After the reaction, we analyzed RNase T_1_ digests of the substrate tRNA^Thr3^(GGU) by LC/MS. In the presence of AdoMet, m^6^t^6^A-containing tetramer (Um^6^t^6^AAGp) could be clearly detected (Figure 3B), whereas no methylation occurred in the absence of AdoMet (Figure 3B). This result demonstrated that YaeB is an AdoMet-dependent methyltransferase responsible for the *N*^6^-methylation of t^6^A at position 37. To reflect this function, we renamed the protein TrmO, according to the preferred nomenclature. When tRNA^Thr3^(GGU) without t^6^A37 was used as the substrate, no methylation was observed (Figure 3C), indicating that the *N*^6^-threonylcarbamoyl moiety of t^6^A37 is definitively required for *N*^6^-methylation by TrmO, even though t^6^A37 is not required for recognition of the tRNA by this enzyme (Figure 3A).

We next examined the *in vitro N*^6^-methylation of native tRNA^Thr3^(GGU) isolated from Δ*trmO*. The isolated tRNA^Thr3^(GGU) that we used for *in vitro* reaction had both t^6^A and ct^6^A at position 37 due to spontaneous hydrolysis of ct^6^A to t^6^A during handling of tRNA. Consequently, m^6^t^6^A was formed in response to a reduction in t^6^A (Figure 3D), whereas ct^6^A did not change upon *in vitro* methylation (Figure 3D), indicating that TrmO does not methylate ct^6^A. Thus, TrmO methylates t^6^A to form m^6^t^6^A before cyclization of t^6^A catalyzed by TcdA.

### Distribution of TrmO and AdoMet-binding site in the β-barrel–type RNA methyltransferase

To elucidate the evolutionary distribution of TrmO homologs, we utilized the genome sequences available in SEED (58). Of the 955 genomes in that database, 318 genomes from all three domains of life contained at least one gene encoding a protein annotated as YaeB (Supplementary Table S1 and http://tinyurl.com/m6t6A37). A summary tree, pruned to the level of taxonomic orders (Figure S4), illustrates that *yaeB* is distributed across all three domains. Bacterial *yaeB* is widely distributed in proteobacteria, especially γ-proteobacteria, all species in Vibrionales, and some species in Firmicutes, but is not present in Lactobacillales or *Mycoplasma* spp. In eukaryotes, *yaeB* homologs are found in vertebrates, insects, and plants, but not in nematodes or fungi. In archaeal phyla, *yaeB* homologs are present in both Euryarchaeota and Crenarchaeota. Multiple alignment of TrmO homologs from representative species revealed a high degree of conservation in the N-terminal region (UPF0066) (Figure 3E and Supplementary Figure S5). The mammalian homologs contain large internal insertions (Supplementary Figure S5).

According to the crystal structures of *Archaeoglobus fulgidus* YaeB in complex with AdoMet (Figure 3F and Supplementary Figure S3)(63), Gln22 (Gln25 in *E. coli*) and Asp111 (Asp125 in *E. coli*) recognize the α-amino group of AdoMet, whereas Arg82 (Arg92 in *E. coli*) and Lys122 (Lys136 in *E. coli*) make hydrogen bonds with the carboxy group of AdoMet. These residues are highly conserved among TrmO homologs (Supplementary Figure S5). To confirm importance of these conserved residues at the AdoMet-binding site, each of the residues was mutated to Ala in a plasmid-encoded *E. coli trmO*. The mutant plasmids were introduced into the Δ*trmO* strain, and total nucleosides extracted from each transformant were analyzed by LC/MS (Figure 3G). Wild-type *trmO* fully rescued m^6^t^6^A formation in Δ*trmO*, whereas the R92A and K136A mutants did not, and m^6^t^6^A formation was also dramatically reduced in the Q25A and D125A mutants. The results suggest that these residues play important roles in AdoMet binding, and that Arg92 and Lys136 in particular are critical residues in TrmO.

Among the γ-proteobacteria, of which *E. coli* is a member, the crystal structure of *Haemophilus influenzae* YaeB homolog has been solved (Figure S3). In addition to the N-terminal β-barrel methyltransferase domain, *H. influenzae* YaeB has an additional C-terminal domain containing the conserved sequence motif, DPRxxY (Figure 3E and Supplementary Figure S5). This domain is specific to YaeB homologs from γ- and β-proteobacteria and mammals (Supplementary Figure S5). Mutation of the conserved motif in the C-terminal domain (D194A/R196A) abolished m^6^t^6^A formation (Figure 3G), indicating that the DPRxxY motif is required for the *N*^6^-methylation reaction.

### TrmO discriminates tRNAs^Thr^ for ACY codons from other isoacceptors

Among the four tRNA^Thr^ isoacceptors in *E. coli*, m^6^t^6^A37 is only present in tRNA^Thr1^(GGU) and tRNA^Thr3^(GGU) (Supplementary Figure S2), both of which have GGU anticodons responsible for ACY codons. TrmO discriminates these ACY-specific tRNAs^Thr^ from other isoacceptors. To determine the elements embedded in these tRNAs that are recognized by TrmO, we performed mutation studies using *in vitro* transcribed tRNAs with enzymatically introduced t^6^A37. By comparing the four isoacceptors, we identified the bases that are common to tRNA^Thr1^(GGU) and tRNA^Thr3^(GGU), but different in the other two isoacceptors (Figure 4A and Supplementary Figure S2); U3-A71, G26, the anticodon stem, G34 (wobble position), and A52-U62. In a control experiment, TrmO efficiently methylated tRNA^Thr3^(GGU), but did not recognize tRNA^Thr4^(UGU) (Figure 4B). Next, we tested a series of tRNA^Thr3^(GGU) variants in which these bases were replaced by the corresponding bases of tRNA^Thr4^(UGU). A variant AS4, in which the anticodon stem was swapped with that of tRNA^Thr4^(UGU), lost the ability to be methylated by TrmO (Figure 4B). In addition, we replaced each of base pairs in the anticodon stem (Figure 4B). When C31-G39 was replaced by A31-U39, little methylation was observed, although modification of other base pairs in the anticodon stem did not influence methylation activity, indicating that TrmO recognizes the bottom base pair (C31-G39) in the anticodon stem of tRNA^Thr1,3^. When G34 was replaced with C or U, a significant reduction in methylation was observed (Figure 4B), indicating that the wobble base G34 acts as another determinant for TrmO. Other differences between tRNA^Thr1,3^ and tRNA^Thr2,4^ did not affect methylation activity. To confirm that C31-G39 and G34 in tRNA^Thr1,3^ act as positive determinants for *N*^6^-methylation by TrmO, we examined tRNA^Thr4^(UGU) variants transplanted with these determinants (Figure 4B). TrmO did not methylate the variant A31C/U39G, in which A31-U39 was replaced by C31-G39, nor the variant U34G, whose wobble U base was replaced by G34. On the other hand, when both determinants were introduced simultaneously (A31C/U39G/U34G), this mutant tRNA^Thr4^(UGU) was methylated by TrmO. In addition, when the entire anticodon stem-loop of tRNA^Thr4^(UGU) was replaced by that of tRNA^Thr3^(GGU) (ASL3), methylation activity increased slightly. From these results, we conclude that C31-G39 and G34 are major determinants of m^6^t^6^A formation, and that other elements in the anticodon stem-loop slightly contribute to efficient methylation by TrmO.

Other ct^6^A-containing tRNAs, tRNA^Ile1^(GAU) and tRNA^Ser3^(GCU), harbor C31-G39 and G34. To determine why these tRNAs cannot serve as substrates of TrmO, we extended the mutation study. The second base of the anticodon (position 35) of these tRNAs differs from that of tRNA^Thr1,3^: tRNA^Ile1^(GAU) and tRNA^Ser3^(GCU) have A35 and C35, respectively; therefore, we constructed tRNA^Thr3^(GGU) variants with either the G35A or G35C mutation. Neither variant was significantly methylated (Figure 4B), showing that TrmO can distinguish tRNA^Thr1,3^ from tRNA^Ile1^(GAU) or tRNA^Ser3^(GCU) by recognizing G35.

### *N* ^6^-methylation of t^6^A enhances the decoding ability of tRNA^Thr^

The *thr* operon is attenuated by efficient translation of the leader peptide encoded by *thrL* (64). Björk and colleagues showed that attenuation of the *thr* operon was alleviated in the *tsaA* mutant (41). Because the ThrL peptide contains consecutive ACC codons (Figure 5A), reduction in the decoding ability of tRNA^Thr1,3^ causes ribosomes to get stuck on this mRNA, thereby preventing transcription termination by unwinding the terminator helix in the 5′ region of the structural genes of the operon. In the *tsaA* mutant, tRNA^Thr1,3^ lacking *N*^6^-methylation at position 37 relieved attenuation of the *thr* operon reporter construct, suggesting that a lack of *N*^6^-methylation decreases the decoding ability of tRNA^Thr1,3^. However, according to the results described above (Figure 2B), m^6^t^6^A of tRNA^Thr1,3^ in the *tsaA* mutant should be converted to ct^6^A, not to t^6^A. Therefore, Qian et al. (41) compared decoding efficiencies of m^6^t^6^A37 versus ct^6^A37 in tRNA^Thr1,3^. To confirm the effect of *N*^6^-methylation of m^6^t^6^A on the decoding ability of tRNA^Thr1,3^, we constructed a reporter consisting of the *thrL* attenuator followed by the firefly luciferase gene (ThrL-Luc) and a control reporter without the attenuator (Luc) (Figure 5B). Each of these reporters was introduced into four *E. coli* strains (WT, Δ*trmO*, Δ*tcdA* and Δ*trmO*/Δ*tcdA*), and the luciferase activities were measured. In the presence of the *thrL* attenuator, Δ*trmO*, in which m^6^t^6^A was converted to ct^6^A, exhibited a 1.5-fold increase in luciferase activity relative to WT (Figure 5C). This result is consistent with the previous report (41). Based on this result, we conclude that m^6^t^6^A facilitates ACC decoding more than ct^6^A. Furthremore, the Δ*trmO*/Δ*tcdA* double-deletion strain, in which m^6^t^6^A was converted to t^6^A, exhibited a 2-fold increase in luciferase activity relative to Δ*tcdA* (Figure 5C). This result clearly demonstrated that the decoding activity of tRNA^Thr1,3^ is reinforced by *N*^6^-methylation of m^6^t^6^A. In addition, comparison of the results from Δ*trmO* and Δ*trmO*/Δ*tcdA* revealed that ct^6^A confers more efficient decoding of ACC codons than t^6^A.

### The human homolog TRMO is responsible for m^6^t^6^A formation of tRNA^Ser^

m^6^t^6^A is present in rat cytoplasmic tRNA^Ser^(GCU) responsible for the AGY codons (34)(Figure 1B). We also detected the m^6^t^6^A nucleoside (*m/z* 427) in total RNA from HeLa cells (Figure 6A), clearly confirming that m^6^t^6^A is actually present in human cells. Then, we isolated tRNA^Ser^(GCU) responsible for AGY codon from total RNA of HeLa cell, analyzed RNase T_1_-digested RNA fragments by LC/MS. As expected, m^6^t^6^A-containing fragments [CUm^6^t^6^AAΨCCAUU(m)Gp] along with t^6^A-containing fragment (CUt^6^AAΨCCAUUGp) and unmodified fragment (CUAAΨCCAUUGp) were clearly observed (Figure 6B). m^6^t^6^A37 was found in 56% of tRNA^Ser^(GCU), while the remaining tRNAs have t^6^A37 (16%) and A37 (16%). The presence of m^6^t^6^A at position 37 was confirmed by collision-induced dissociation (CID) (Figure 6C).

To identify the human enzyme responsible for *N*^6^-methylation of m^6^t^6^A, we searched for homologs of *E. coli* TrmO, and retrieved C9orf156 as a plausible human homolog (Supplementary Figure S5). In the NCBI database, C9orf156 was misannotated as nef-associated protein 1 (NAP1), which should be annotated as ACOT8. C9orf156 shares 41% sequence identity with *E. coli* TrmO. To determine whether this gene is responsible for *N*^6^-methylation of m^6^t^6^A in human tRNA, we recombinantly expressed C9orf156 in *E. coli* and purified it to homogeneity. To generate the substrate, we prepared human tRNA^Ser^(GCU) transcript containing t^6^A37 introduced *in vitro* using *E. coli* t^6^A-modifying enzymes (Figure 6D). As shown in Figure 6E, m^6^t^6^A-containing trimer (m^6^t^6^AAUp) was clearly detected only in the presence of both recombinant C9orf156 and AdoMet. We also confirmed m^6^t^6^A formation by LC/MS nucleoside analysis (Figure 6F). Based on this protein's homology to *E. coli* TrmO and its similar biochemical function, we named it TRMO. Additionally, we investigated whether *E. coli* TrmO could use human tRNA^Ser^(GCU) as a substrate, but detected no methylation (Figure 6E and F), possibly because human tRNA^Ser^(GCU) has A31-U39 and C35, which should function as anti-determinants for *E. coli* TrmO. These results indicated that the substrate recognition mechanism of human TRMO differs from that of *E. coli* TrmO.

## DISCUSSION

To date, seven classes of AdoMet-dependent methyltransferases have been described (7,8,65). TrmO/YaeB has a single-sheeted β-barrel structure encoded by the conserved UPF0066 domain, and does not belong to any known classes of AdoMet-dependent methyltransferases. Class V AdoMet-dependent methyltransferase also has an anti-parallel β-barrel structure composed of four β-sheets in a SET domain (Supplementary Figure S6)(66–68). The class V methyltransferases, which contain the SET domain, participate in Lys-methylation of histones, a process involved in chromatin remodeling (1,66). The conserved motifs in the SET domain constitute a pseudoknot structure (69), which configures the AdoMet binding pocket with a site for the target Lys residue. However, TrmO/YaeB does not have any of the motifs or pseudoknot structures conserved in the SET domain (Supplementary Figures S3 and S6A). In addition, the β-barrel of TrmO/YaeB is composed of a single sheet of six consecutive β-sheets (Supplementary Figure S6B), whereas the β-barrel of SET domain is composed of four β-sheets (Supplementary Figure S6A). Therefore, TrmO belongs to a novel class of AdoMet methyltransferase, designated as class VIII.

In *E. coli*, YfiC (TrmM or TrmN6) is another *N*^6^-methyltransferase responsible for m^6^A formation at position 37 in tRNA^Val^ (UAC) (70). Although there was a possibility that YfiC/TrmM redundantly *N*^6^-methylates t^6^A in tRNA^Thr1,3^, we did not observe any m^6^t^6^A in Δ*trmO* strain (Figure 2AB), showing that YfiC/TrmM is not involved in m^6^t^6^A formation. YfiC/TrmM harbors a motif termed Methyltransferase_26 which contains a catalytic site of TaqI DNA *N*^6^ adenosine methyltransferase, categorized in class I AdoMet-dependent methyltransferase (8,71,72). Therefore, TrmO and YfiC/TrmM have evolved from distinct ancestors, albeit both enzymes catalyze *N*^6^-methylation of A37 of tRNAs.

In the three YaeB homologs for which crystal structures are available, the AdoMet binding pockets are almost identical (Supplementary Figure S7). AdoMet binds to the upper part of the β-barrel with surrounding extra loops (Supplementary Figures S3 and S6B). In our mutational study of *E. coli* TrmO, we examined four residues (Gln25, Arg92, Asp111, and Lys136) that are likely to be involved in AdoMet binding, according to the crystal structure of *A. fulgidus* YaeB (AF0241). Both Arg92 and Lys136, which we demonstrated to be critical for *N*^6^-methylation of t^6^A, recognize the carboxyl group of AdoMet. Intriguingly, the side chain of Lys122 (Lys136 in *E. coli*) in *A. fulgidus* YaeB extends from the other monomer and interacts with the carboxyl group of AdoMet (Figure 3F and Supplementary Figure S7)(63). This intersubunit composition of the AdoMet-binding pocket is a unique feature of class VIII AdoMet methyltransferases.

In bacteria and eukaryotes, TrmO has an additional C-terminal domain containing the conserved DPRxxY motif. We found that Asp194 and Arg196 in this motif of *E. coli* TrmO are necessary for *N*^6^-methylation. Many RNA methyltransferases have RNA-binding domains, including THUMP (49,73), the OB-fold (74), and the PUA domain (75), in addition to the catalytic domains in complex with AdoMet. Thus, we hypothesize that the C-terminal domain of TrmO plays a role in tRNA binding.

According to phylogenetic analysis, *yaeB* homologs are widely distributed in Archaea. Several archaea contain multiple homologs of *yaeB*, e.g., *Methanoculleus marisnigri* contains seven *yaeB* paralogs (Supplementary Table S1). However, no archaeal YaeB has a C-terminal domain containing the DPRxxY motif that is conserved in bacterial and mammalian TrmO homologs. In addition, m^6^t^6^A has never been detected in any archaeal tRNAs analyzed so far (34). *Halobacterium salinarum* is one of the few archaea in which the tRNA modifications have been characterized, but m^6^t^6^A was not detected in tRNA^Thr^ responsible for ACY codons (76), even though a YaeB homolog is present in *Halobacterium salinarum* strain NRC-1 (Supplementary Table S1). Thus, archaeal YaeB might not be involved in m^6^t^6^A formation, but is a class VIII AdoMet methyltransferase that might target another RNA or protein for methylation. However, we cannot exclude the possibility that m^6^t^6^A is present in other unanalyzed tRNAs from archaeal species whose genomes encode YaeB homologs. A Bayesian analysis showed that a branch of bacterial YaeB is intermingled with the archaeal branch (Supplementary Figure S8). Although very few bacteria contain multiple YaeB paralogs (Supplementary Table S1), *Shewanella halifaxensis* HAW-EB4 and *Desulfotalea psychrophila* LSv54 each have two paralogs of YaeB that are phylogenetically distant, and one paralog from each species is grouped within the archaeal branch.

In this study, we showed that m^6^t^6^A has a stronger ability to attenuate the *thrL* operon than ct^6^A and t^6^A, indicating that *N*^6^-methylation of t^6^A reinforces the decoding ability of tRNA^Thr1,3^. In addition, ct^6^A conferred more efficient ACC decoding than t^6^A. Previously, we showed that cyclization of t^6^A is an additional modification that supports the decoding efficiency of tRNA^Lys^ (31). Taken together with the results in this study, ct^6^A supports the decoding efficiency of other tRNAs in general. To discuss the molecular basis of decoding ability by *N*^6^-methylation of t^6^A, we constructed a structural model of m^6^t^6^A at the ribosomal A-site, based on the crystal structure of the anticodon stem-loop with t^6^A recognizing the AAG codon (11) (Supplementary Figure S9A and B). The *N*^6^-methyl group of m^6^t^6^A37 occupied the space between the carbonyl oxygen (O4) of U36 and the exocyclic amine (N6) of A38 (Supplementary Figure S9B). The distances from the *N*^6^-methyl group to O4 of U36 and to N6 of A38 were 3.3 Å and 3.2 Å, respectively. These distances are close enough to form van der Waals interactions, which would strengthen the stacking ability of m^6^t^6^A37 with U36 and A38. In solution structure, t^6^A37 destabilizes the anticodon stacking by promoting U36 bulges (77), implying that the *N*^6^-methyl group might have a function in stabilizing the anticodon by preventing the fluctuation of the uracil base of U36 at the ribosomal A-site. On the other hand, ct^6^A37 strengthens recognition of the first adenosine base of ANN codons, due to increased stacking and the additional hydrogen bond (Supplementary Figure S9C) (Miyauchi et al., 2013). We hypothesize that ct^6^A and m^6^t^6^A increase the decoding abilities of tRNAs via distinct mechanisms.

**Figure 7.**
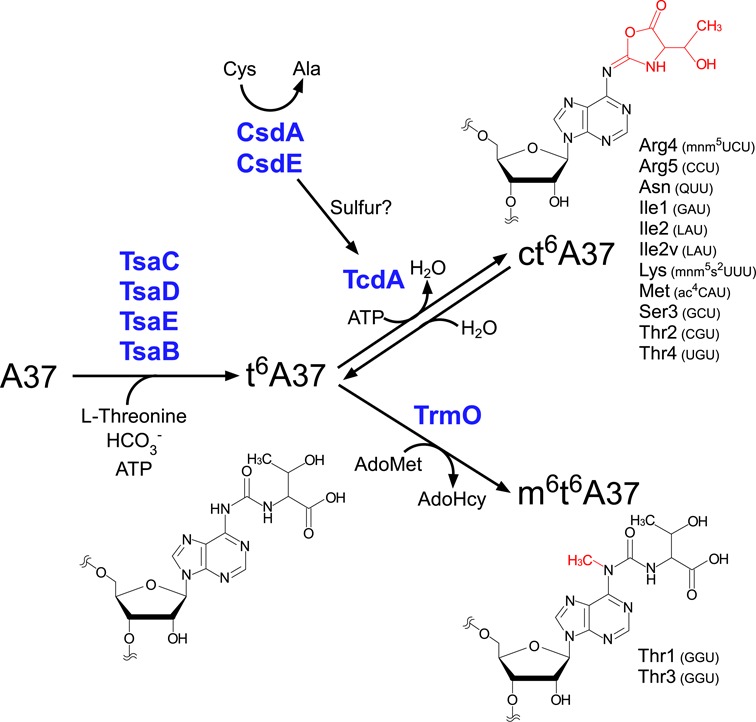
Biosynthesis of t^6^A derivatives. In *E. coli*, A37 of all 13 tRNAs responsible for ANN codons is modified to t^6^A by four enzymes (YgjD, YjeE, YeaZ and YrdC), which use *L*-threonine, bicarbonate, and ATP as substrates. For 11 tRNAs (i.e., excluding tRNA^Thr1,3^), t^6^A37 is further dehydrated to ct^6^A by TcdA in the presence of ATP. This cyclization reaction is activated by cysteine desulfurase CsdA and sulfur acceptor protein CsdE. For tRNA^Thr1,3^, t^6^A37 is methylated to form m^6^t^6^A37; this reaction is catalyzed by TrmO using AdoMet as a methyl donor.

Biogenesis of m^6^t^6^A is depicted in Figure 7. There are two t^6^A derivatives in *E. coli* tRNAs: 11 species of tRNAs responsible for ANN codons have ct^6^A37, whereas tRNAs^Thr1,3^ have m^6^t^6^A37. Initially, t^6^A is formed at position 37 on tRNAs by four enzymes, TsaB, TsaC, TsaD and TsaE, using *L*-threonine, bicarbonate, and ATP as substrates (21). Thereafter, TrmO methylates t^6^A in tRNA^Thr1,3^ to form m^6^t^6^A, using AdoMet as the methyl donor. TrmO specifically recognizes the bottom base pair (C31–G39) of the anticodon stem and G34 as positive determinants to select these tRNAs. TrmO cannot employ ct^6^A as a substrate to form m^6^t^6^A. On the other hand, TcdA catalyzes ATP-dependent dehydration of t^6^A to form ct^6^A in 11 tRNAs responsible for ANN codons (31). This reaction is activated by the cysteine desulfurase CsdA and the sulfur-transfer protein CsdE (31), indicating that the sulfur-relay system is involved in efficient ct^6^A formation. In the absence of TrmO, t^6^A in tRNA^Thr1,3^ is also converted to ct^6^A. This observation suggests that t^6^A is a common precursor of ct^6^A and m^6^t^6^A. In addition, TcdA has the ability to recognize all 13 tRNA species bearing t^6^A37, including tRNAs^Thr1,3^, and form ct^6^A in these species. In other words, tRNA^Thr1,3^ do not have any anti-determinants for TcdA. Hence, TrmO competes against TcdA for recognition of tRNA^Thr1,3^ as a target for m^6^t^6^A formation. If t^6^A dehydration and ct^6^A hydrolysis are in equilibrium in the cell, TrmO might shift the equilibrium toward t^6^A by catching tRNA^Thr1,3^ bearing t^6^A, leading to the accumulation of m^6^t^6^A.

In human cells, we determined that TRMO (C9orf156) is responsible for this methylation. Because little information on this gene is available, the physiological role of m^6^t^6^A in human tRNAs remains unknown. According to their different substrate specificities, bacterial TrmO and mammalian TRMO should have evolved differently in their respective species to employ specific tRNAs as substrates, optimizing the translational efficiency and fidelity in the physiological context of each organism. In contrast to bacterial tRNAs, mammalian tRNAs do not possess ct^6^A37, suggesting that *N*^6^-methylation of t^6^A has a profound effect on the decoding ability of tRNA^Ser^(GCU). In this study, we showed that m^6^t^6^A is partially introduced in tRNA^Ser^(GCU) in HeLa cell (Figure 6B), implying the frequency of m^6^t^6^A is regulated. In the human proteome, Ser-rich sequences can be found in RS domains in SR proteins, which are key factors in RNA splicing (78). Thus, it is tempting to speculate that m^6^t^6^A in tRNA^Ser^(GCU) can modulate the translational efficiency of AGY-encoded Ser clusters in SR proteins, suggesting an intriguing regulatory mechanism of translational efficiency mediated by the frequency of m^6^t^6^A.

## SUPPLEMENTARY DATA

Supplementary Data are available at NAR Online.

SUPPLEMENTARY DATA
