# Supplementary material for: Discovery of the β-barrel–type RNA methyltransferase responsible for N6-methylation of N6-threonylcarbamoyladenosine in tRNAs
Source: Nucleic Acids Res. 2014 Jul 24;42(14):9350–65. doi: 10.1093/nar/gku618 (PMC4132733; doi:10.1093/nar/gku618)
Supplement: SUPPLEMENTARY DATA [file supp_42_14_9350__index.html]

Discovery of the β-barrel–type RNA methyltransferase responsible for N6-methylation of N6-threonylcarbamoyladenosine in tRNAs — Discovery of the β-barrel–type RNA methyltransferase responsible for N6-methylation of N6-threonylcarbamoyladenosine in tRNAs — SUPPLEMENTARY DATA 

# Discovery of the β-barrel–type RNA methyltransferase responsible for *N*6-methylation of *N*6-threonylcarbamoyladenosine in tRNAs

## SUPPLEMENTARY DATA

**Files in this Data Supplement:**

- SUPPLEMENTARY DATA
- SUPPLEMENTARY DATA
- SUPPLEMENTARY DATA
- SUPPLEMENTARY DATA
- SUPPLEMENTARY DATA
- SUPPLEMENTARY DATA
- SUPPLEMENTARY DATA
- SUPPLEMENTARY DATA
