# Supplementary material for: Discovery of the β-barrel–type RNA methyltransferase responsible for N6-methylation of N6-threonylcarbamoyladenosine in tRNAs
Source: Nucleic Acids Res. 2014 Jul 24;42(14):9350–65. doi: 10.1093/nar/gku618 (PMC4132733; doi:10.1093/nar/gku618)
Supplement: SUPPLEMENTARY DATA [file supp_gku618_nar-01435-r-2014-File004.pdf]

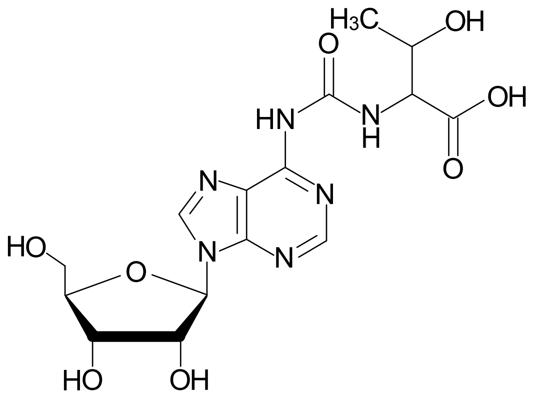

N^6-threonylcarbamoyladenosine (t^6A)

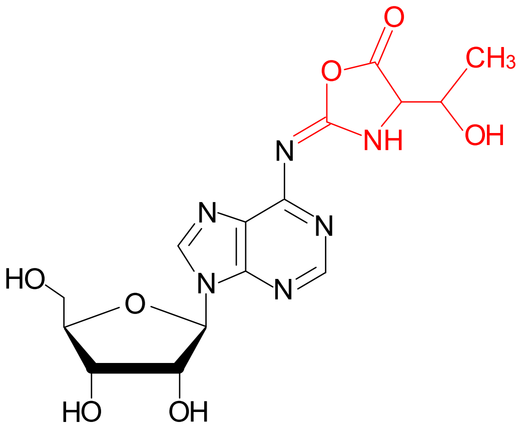

Cyclic-N^6-threonylcarbamoyladenosine (ct^6A)

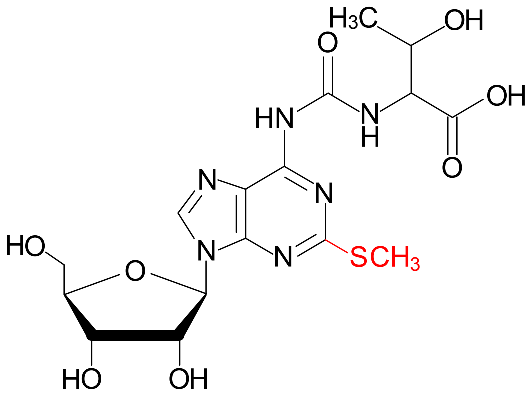

2-methylthio-N^6-threonylcarbamoyladenosine (ms^2t^6A)

Figure S1

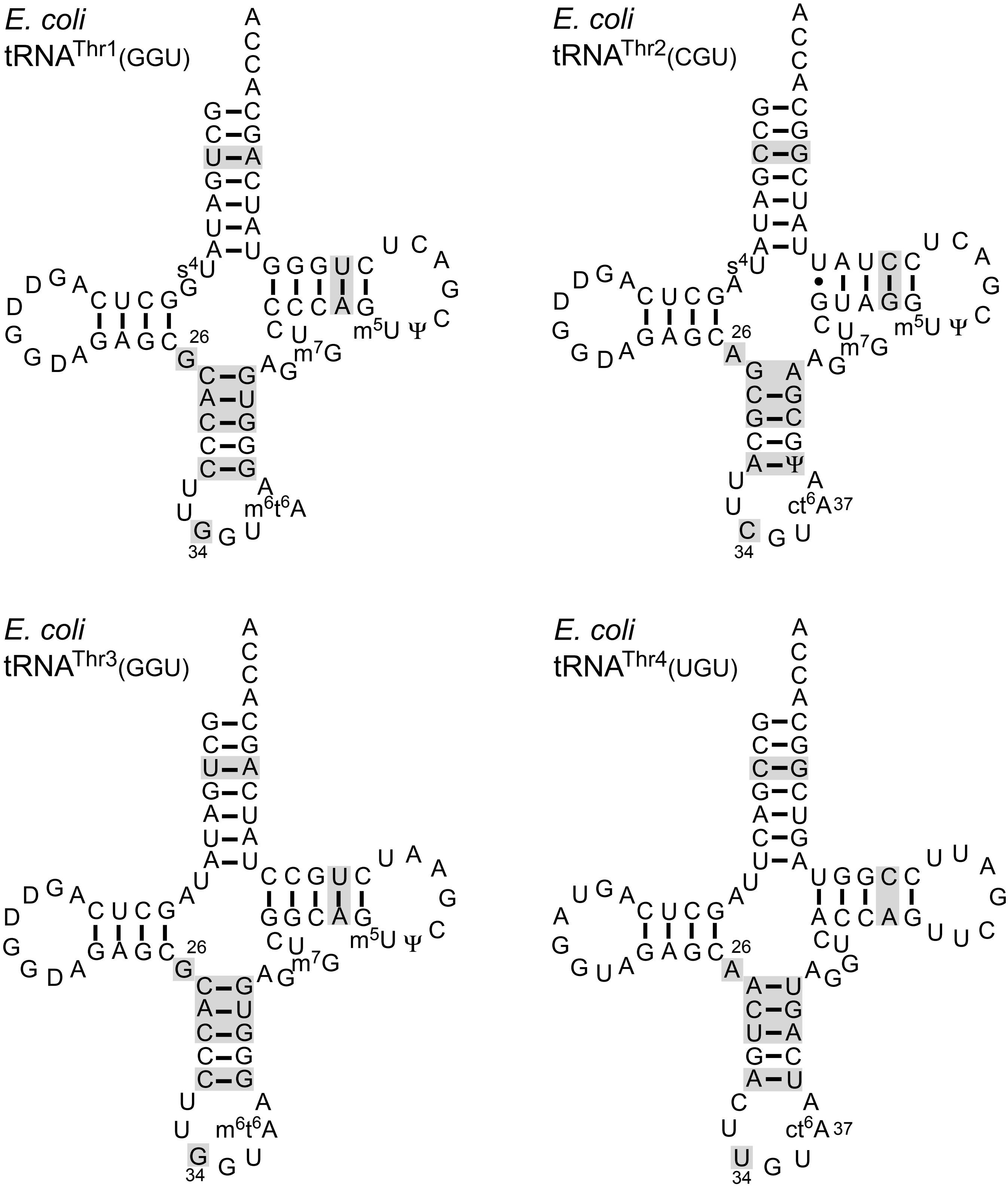
E. coli
tRNAThr1(GGU)
E. coli
tRNAThr2(CGU)
E. coli
tRNAThr3(GGU)
E. coli
tRNAThr4(UGU)

Figure S2

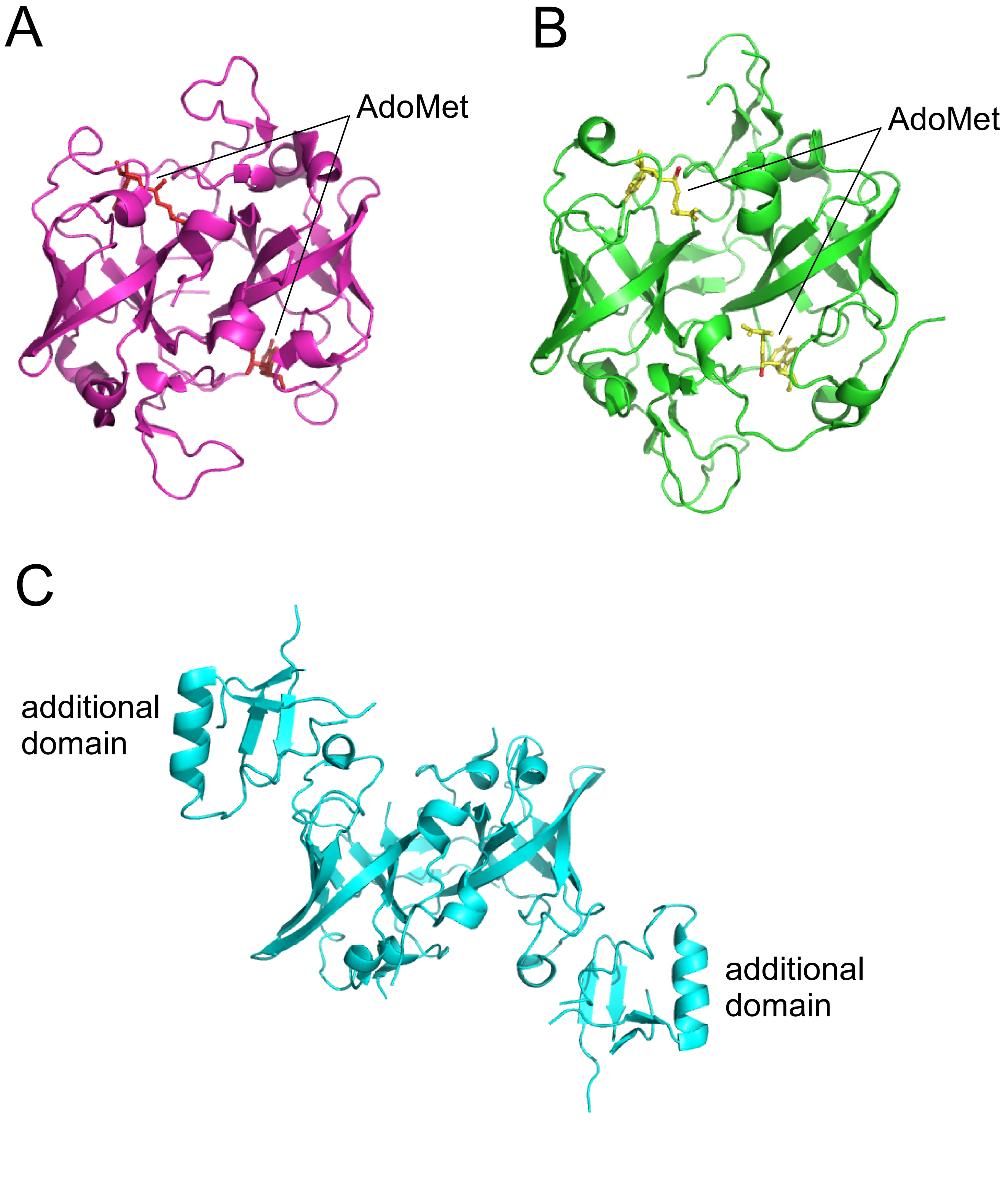

Figure S3

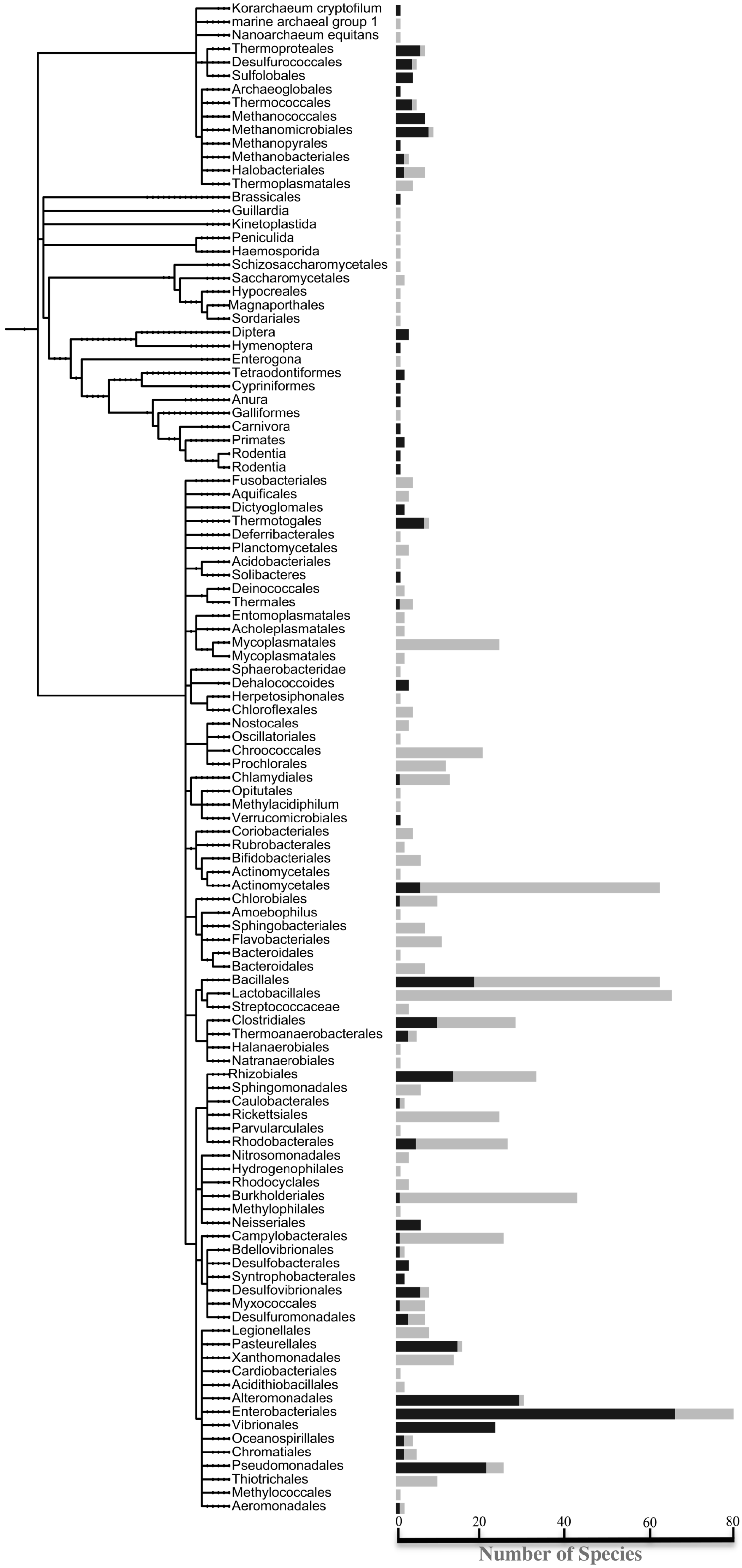
Korarchaeum cryptofilum
marine archaeal group 1
Nanoarchaeum equitans
Thermoproteales
Desulfurococcales
Sulfolobales
Archaeoglobales
Thermococcales
Methanococcales
Methanomicrobiales
Methanopyrales
Methanobacteriales
Halobacteriales
Thermoplasmatales
Brassicales
Guillardia
Kinetoplastida
Peniculida
Haemosporida
Schizosaccharomycetales
Saccharomycetales
Hypocreales
Magnaporthales
Sordariales
Diptera
Hymenoptera
Enterogona
Tetraodontiformes
Cypriniformes
Anura
Galliformes
Carnivora
Primates
Rodentia
Rodentia
Fusobacteriales
Aquificales
Dictyoglomales
Thermotogales
Deferribacterales
Planctomycetales
Acidobacteriales
Solibacteres
Deinococcales
Thermales
Entomoplasmatales
Acholeplasmatales
Mycoplasmatales
Mycoplasmatales
Sphaerobacteridae
Dehalococcoides
Herpetosiphonales
Chloroflexales
Nostocales
Oscillatoriales
Chroococcales
Prochlorales
Chlamydiales
Opitutales
Methylacidiphilum
Verrucomicrobiales
Coriobacteriales
Rubrobacterales
Bifidobacteriales
Actinomycetales
Actinomycetales
Chlorobiales
Amoebophilus
Sphingobacteriales
Flavobacteriales
Bacteroidales
Bacteroidales
Bacillales
Lactobacillales
Streptococcaceae
Clostridiales
Thermoanaerobacterales
Halanaerobiales
Natranaerobiales
Rhizobiales
Sphingomonadales
Caulobacterales
Rickettsiales
Parvularculales
Rhodobacterales
Nitrosomonadales
Hydrogenophilales
Rhodocyclales
Burkholderiales
Methylophilales
Neisseriales
Campylobacterales
Bdellovibrionales
Desulfobacterales
Syntrophobacterales
Desulfovibrionales
Myxococcales
Desulfuromonadales
Legionellales
Pasteurellales
Xanthomonadales
Cardiobacteriales
Acidithiobacillales
Alteromonadales
Enterobacteriales
Vibrionales
Oceanospirillales
Chromatiales
Pseudomonadales
Thiotrichales
Methylococcales
Aeromonadales
0
20
40
60
80
Number of Species

Figure S4

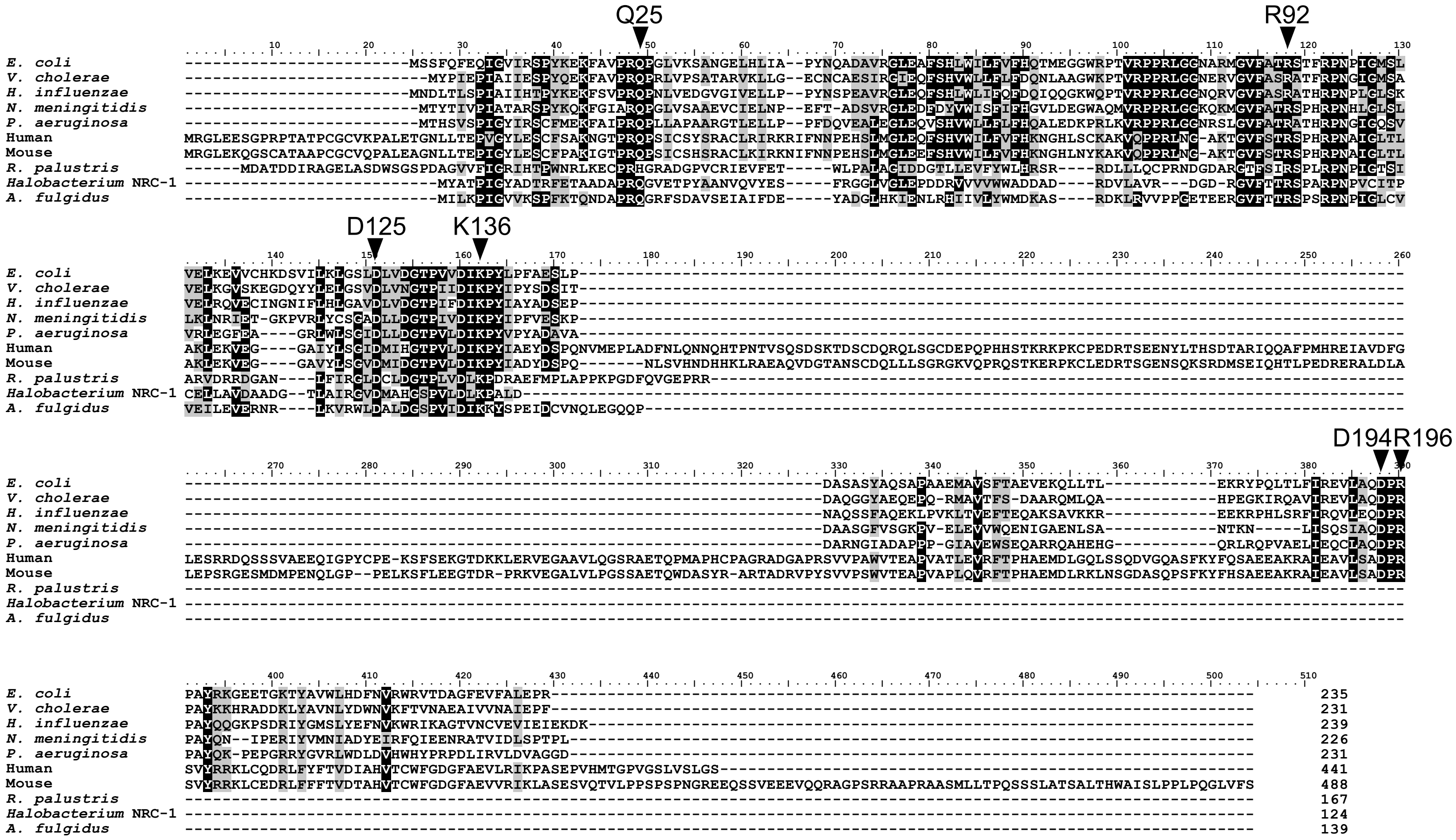
Q25
R92
D125
K136
D194R196
E. coli
V. cholerae
H. influenzae
N. meningitidis
P. aeruginosa
Human
Mouse
R. palustris
Halobacterium NRC-1
A. fulgidus
235
231
239
226
231
441
488
167
124
139

Figure S5

A

Human SETD7

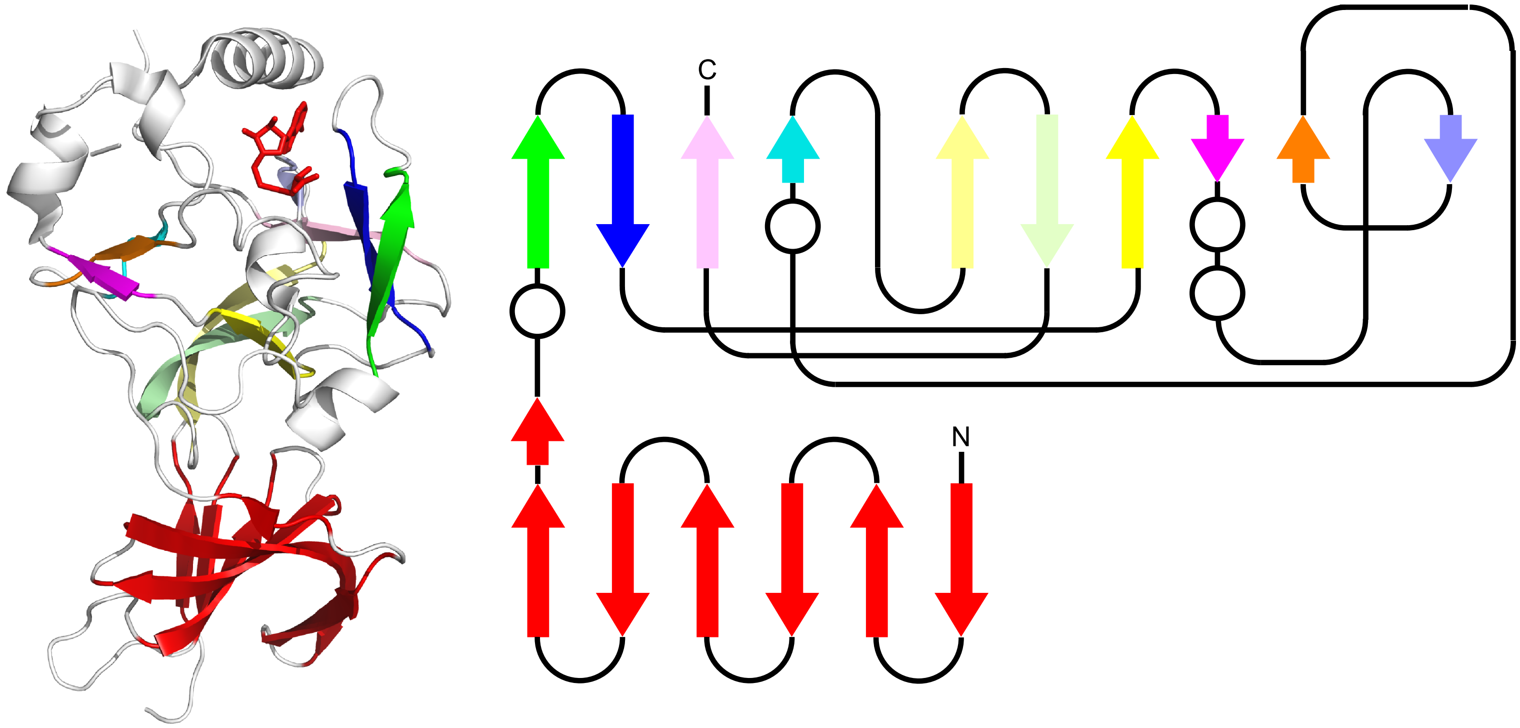

B

A. fulgidus YaeB (AF0241)

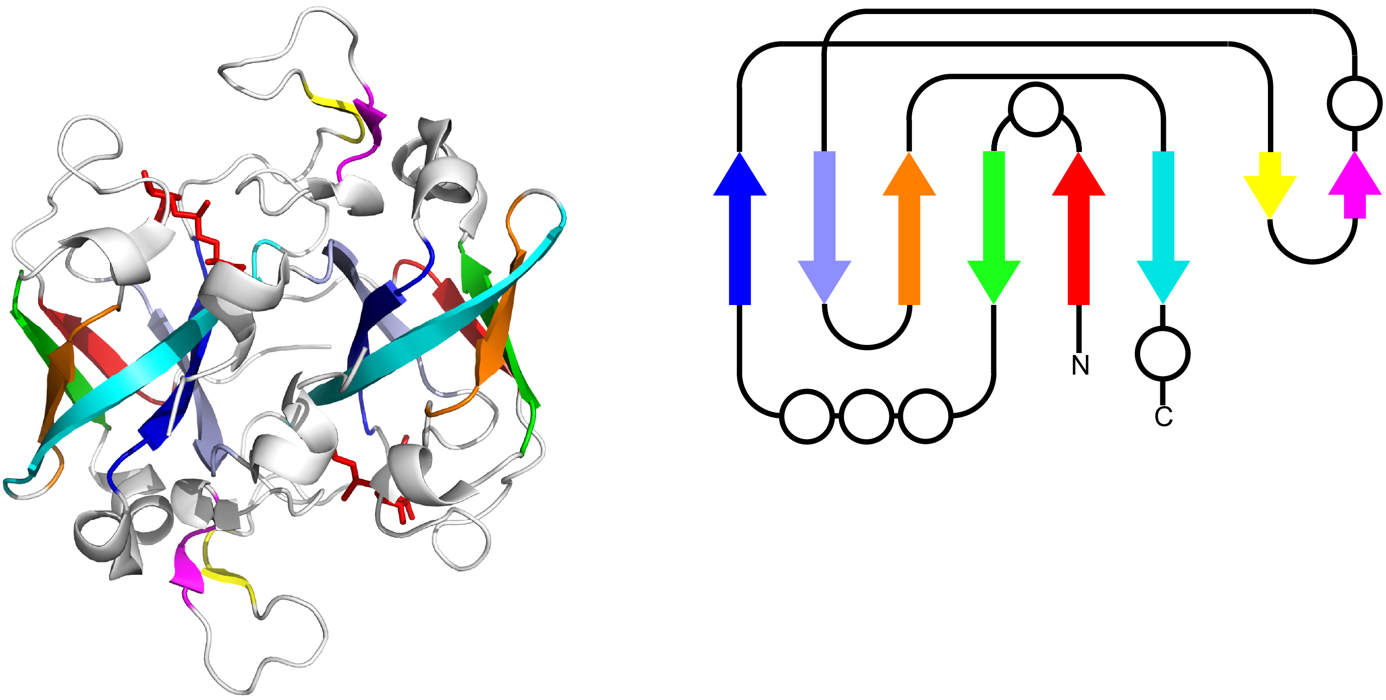

Figure S6

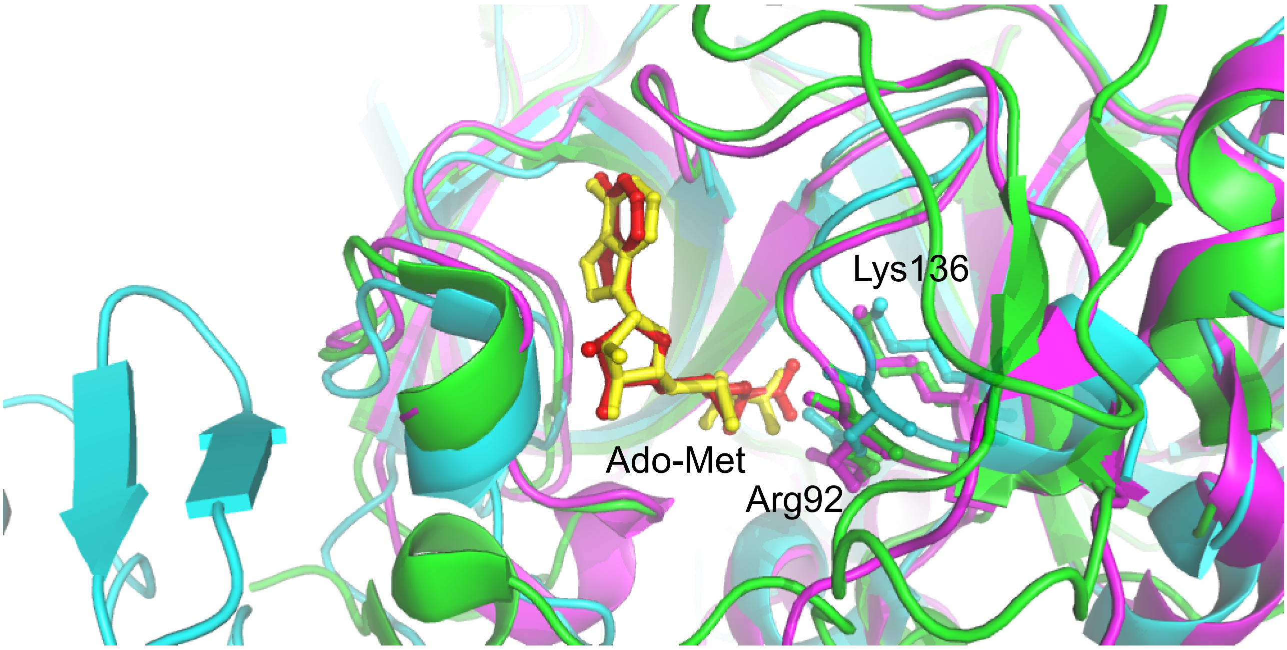

Figure S7

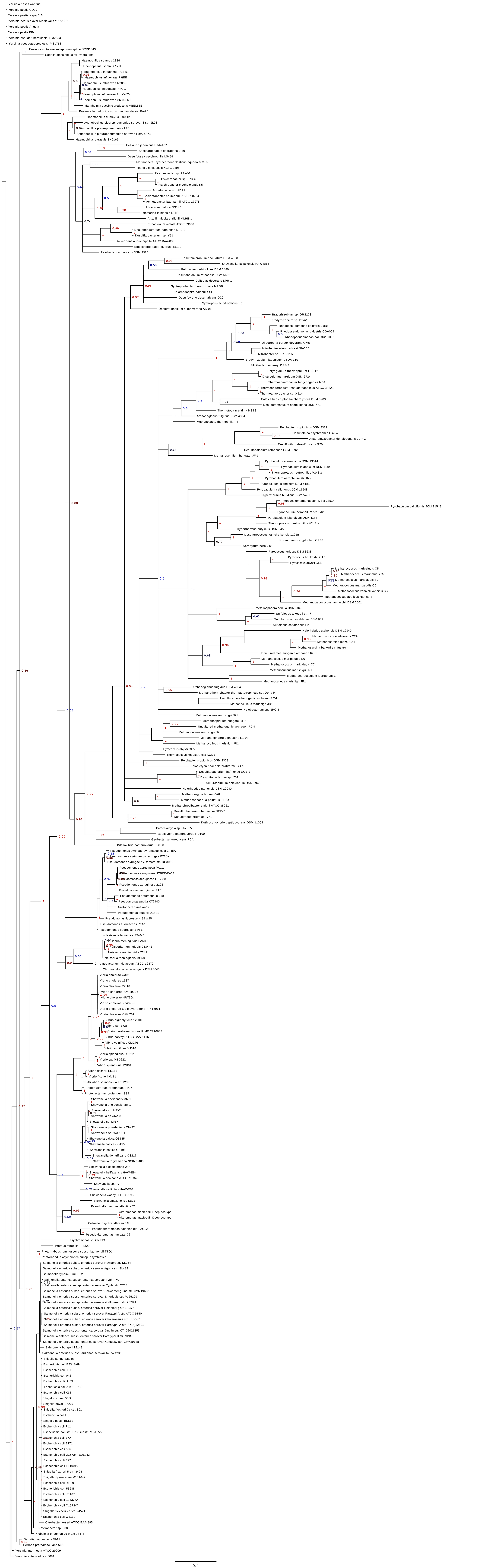
0.4

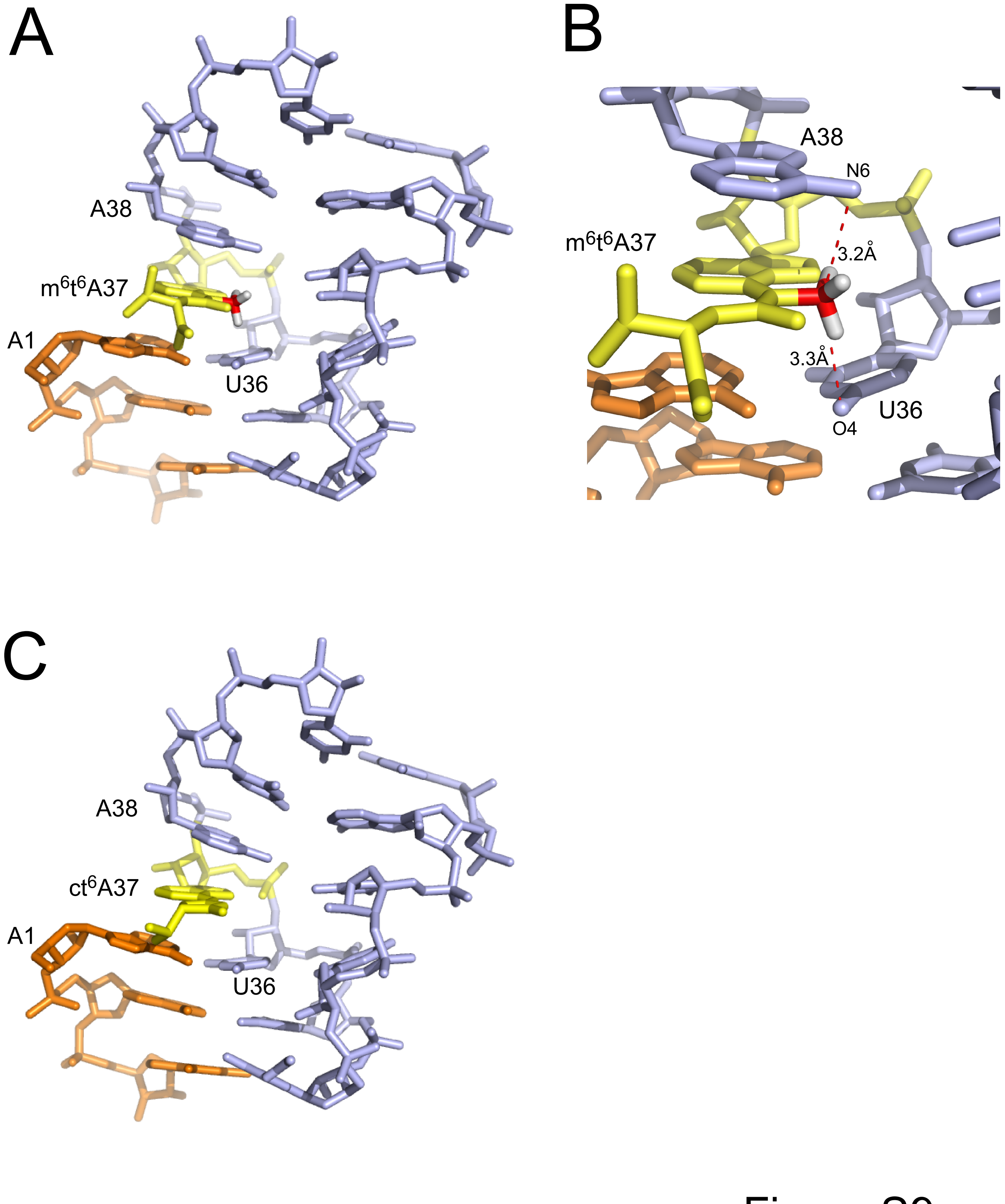
A
A38
m6t6A37
A1
U36
B
A38
N6
m6t6A37
3.2Å
3.3Å
U36
O4
C
A38
ct6A37
A1
U36

Figure S9
