# Supplementary material for: Discovery of the β-barrel–type RNA methyltransferase responsible for N6-methylation of N6-threonylcarbamoyladenosine in tRNAs
Source: Nucleic Acids Res. 2014 Jul 24;42(14):9350–65. doi: 10.1093/nar/gku618 (PMC4132733; doi:10.1093/nar/gku618)
Supplement: SUPPLEMENTARY DATA [file supp_gku618_nar-01435-r-2014-File003.docx]

**Supplementary information**

**Discovery of the β-barrel–type RNA methyltransferase responsible for *N*^6^-methylation of *N*^6^-threonylcarbamoyladenosine in tRNAs**

Kimura et al.

**Supplementary Result**

We mapped the *tsaA* mutation based on the genetic information provided by Qian et al. ([1](#_ENREF_1)). It had been mapped the left of the *zae*-3095::Tn10*kan* with 75% co-transduction frequency between the two alleles. The exact position of the *Zae*-3095::Tn10 marker is now known and corresponds to *yafC*3095::Tn10*kan* at position 230,099 (4. 97 min on the current map)([2](#_ENREF_2)). Using the Wu formula that links physical distance to co-transduction frequency ([3](#_ENREF_3)), the *tsaA* allele maps 9-10 kb to the left of the *yafC* allele at position 220-221kb around 4.72 minutes. The genes located in that region are *proS, yaeB,rcsF and metQ* ([4](#_ENREF_4)). We identified *yaeB* gene, because *yaeB* is the sole gene of unknown function among these genes.

**Legends for Supplementary Figures**

**Figure S1 Chemical structures of t^6^A, ct^6^A, and ms^2^t^6^A**

Structural alterations (relative to t^6^A) are shown in red.

**Figure S2 Secondary structures of *E. coli* tRNAs for Thr1, Thr2, Thr3, and Thr4 with post-transcriptional modifications**

Bases that are common in tRNA^Thr1,3^ but different in tRNA^Thr2,4^ are highlighted in gray in each tRNA. Post-transcriptional modifications are 4-thiouridine (s^4^U), dihydrouridine (D), *N*^6^-methyl-*N*^6^-threonylcarbamoyladenosine (m^6^t^6^A), cyclic-*N*^6^-threonylcarbamoyladenosine (ct^6^A), 7-methylguanosine (m^7^G), 5-methyluridine (m^5^U), and pseudouridine (Ψ). The post-transcriptional modifications of *E. coli* tRNA^Thr4^ are not yet known, but we confirmed the presence of ct^6^A37 in this tRNA (data not shown).

**Figure S3 Crystal structures of YaeB homologs**

Ribbon representation of crystal structures of AF0241 (PDB:2NV4) of *Archaeoglobus fulgidus* in Euryarchaeota (A), RPA0152 (PDB:3OKX) of *Rhodopseudomonas palustris* in α-proteobacteria (B), and HI0510 (PDB:1XQB) of *Haemophilus influenzae* in γ-proteobacteria (C).

**Figure S4 Taxonomic distribution of *yaeB* homologs, pruned to the level of orders**

Occurrence of YaeB in each order. Black represents presence of a homolog, whereas gray represents absence of a homolog; bar lengths correspond to the number of genomes in each category (i.e., present or absent) within each order.

**Figure S5** **Sequence alignment of YaeB homologs of representative organisms**

Amino acid sequences were obtained from the NCBI database, aligned using Clustal X2 ([5](#_ENREF_5)), and displayed using Bioedit (http://www.mbio.ncsu.edu/bioedit/bioedit.html). The following proteins are shown (accession numbers are given in parentheses): *E. coli* TrmO (TsaA) (NP_414737.1), *Vibrio cholerae* VC0876 (NP_230523.1), *Haemophilus influenzae* HI0510 (NP_438668.1), *Neisseria meningitidis Z2491* NMA0245 (YP_002341779.1), *Pseudomonas aeruginosa* PA3388 (NP_252078.1), *Homo sapiens* TRMO (NP_057565.3), *Mus musculus* TRMO (NP_083362.1), *Rhodopseudomonas palustris* RPA0152 (NP_945505.1), *Halobacterium sp. NRC-1* VNG1115H (NP_280026.1), and *Archaeoglobus fulgidus* AF0241 (NP_069079.1).

Positions examined in the mutation study are shown above the alignment.

**Figure S6 Comparison of β-barrel structures of class V and VIII AdoMet methyltransferases**

Crystal structures and topology diagrams of human SETD7 (PDB:3M5A) (A), shown as a representative of class V AdoMet methyltransferases, and A. *fulgidus* AF0241 (PDB:2NV4) (B), shown as a representative of class VIII AdoMet methyltransferases. β-sheets are labeled according to the same color code in the topology diagrams and the ribbon presentations of the crystal structures.

**Figure S7 AdoMet-binding sites of three YaeB homologs**

Crystal structures of AF0241 (magenta), RPA0152 (green), and HI0510 (light blue), compared by the best-fit superposition. The color code is the same as in Supplementary Figure S3. AdoMet, Arg92, and Lys136 are shown in stick representation.

**Figure S8 Phylogenetic analysis of *yaeB* homologs in Archaea and Bacteria**

Sequence alignments, the input nexus file, the output consensus tree with support values are attached as **Supplemental materials**.

**Figure S9 Structural model of m^6^t^6^A in the anticodon stem-loop (ASL), which recognizes the codon in the ribosomal A-site**

A The *N*^6^-methyl group was added to the crystal structure coordinates of the ternary complex of *Thermus thermophilus* 30S ribosomal subunit, mRNA, and ASL^Lys^ (PDB: 1XMO) ([6](#_ENREF_6)). ASL^Lys^ and mRNA are shown in light blue and orange, respectively. m^6^t^6^A is in yellow, and the carbon of the *N*^6^-methyl group is in red.

B Close-up view of the *N*^6^-methyl group in m^6^t^6^A, with the surrounding region. Distances from the *N*^6^-methyl group to O4 of U36 and to N6 of A38 are indicated by dashed lines.

C Structural model of ct^6^A in the ASL, recognizing the codon at the ribosomal A-site ([7](#_ENREF_7)).

**Table S1. Distribution of *yaeB* homologs in 955 genomes in SEED**

**Table S2 List of primers used in this study**

**REFERENCES**

1. Qian, Q., Curran, J.F. and Björk, G.R. (1998) The methyl group of the *N*^6^-methyl-*N*^6^-threonylcarbamoyladenosine in tRNA of *Escherichia coli* modestly improves the efficiency of the tRNA. *J. Bacteriol.*, **180**, 1808-1813.

2. Singer, M., Baker, T.A., Schnitzler, G., Deischel, S.M., Goel, M., Dove, W., Jaacks, K.J., Grossman, A.D., Erickson, J.W. and Gross, C.A. (1989) A collection of strains containing genetically linked alternating antibiotic resistance elements for genetic mapping of *Escherichia coli*. *Microbiol. Rev.*, **53**, 1-24.

3. Wu, T.T. (1966) A model for three-point analysis of random general transduction. *Genetics*, **54**, 405-410.

4. Zhou, J. and Rudd, K.E. (2013) EcoGene 3.0. *Nucleic Acids Res.*, **41**, D613-624.

5. Larkin, M.A., Blackshields, G., Brown, N.P., Chenna, R., McGettigan, P.A., McWilliam, H., Valentin, F., Wallace, I.M., Wilm, A., Lopez, R. *et al.* (2007) Clustal W and Clustal X version 2.0. *Bioinformatics*, **23**, 2947-2948.

6. Murphy, F.V., Ramakrishnan, V., Malkiewicz, A. and Agris, P.F. (2004) The role of modifications in codon discrimination by tRNA(Lys)UUU. *Nat. Struct. Mol. Biol.*, **11**, 1186-1191.

7. Miyauchi, K., Kimura, S. and Suzuki, T. (2013) A cyclic form of *N*^6^-threonylcarbamoyladenosine as a widely distributed tRNA hypermodification. *Nat. Chem. Biol.*, **9**, 105-111.
