# Supplementary material for: Discovery of the β-barrel–type RNA methyltransferase responsible for N6-methylation of N6-threonylcarbamoyladenosine in tRNAs
Source: Nucleic Acids Res. 2014 Jul 24;42(14):9350–65. doi: 10.1093/nar/gku618 (PMC4132733; doi:10.1093/nar/gku618)
Supplement: SUPPLEMENTARY DATA [file supp_gku618_nar-01435-r-2014-File006.docx]

**Table S2. List of primers used in this study.**

Primers used in this study for *in vitro* transcription, cloning, and site-directed mutagenesis are listed.

| **Primers for DNA templates for *in vitro* transcription** | |
| --- | --- |
| Thr3_5prime | GCTAATACGACTCACTATAGCTGATATAGCTCAGTTGGTA |
| Thr3_body | GATATAGCTCAGTTGGTAGAGCGCACCCTTGGTAAGGGTGAGGTCGGCA |
| Thr3_3prime | TGGTGCTGATAGGCAGATTCGAACTGCCGACCTCACCCTT |
| Thr3_AS_ body | GATATAGCTCAGTTGGTAGAGCGACTGATTGGTAATCAGTAGGTCGGCA |
| Thr3_AS_3prime | TGGTGCTGATAGGCAGATTCGAACTGCCGACCTACTGATT |
| Thr3_C27A_G43U_body | GATATAGCTCAGTTGGTAGAGCGAACCCTTGGTAAGGGTTAGGTCGGCA |
| Thr3_A28C_U42G_body | GATATAGCTCAGTTGGTAGAGCGCCCCCTTGGTAAGGGGGAGGTCGGCA |
| Thr3_C29U_G41A_body | GATATAGCTCAGTTGGTAGAGCGCATCCTTGGTAAGGATGAGGTCGGCA |
| Thr3_C30G_G40C_body | GATATAGCTCAGTTGGTAGAGCGCACGCTTGGTAAGCGTGAGGTCGGCA |
| Thr3_C31A_G39U_body | GATATAGCTCAGTTGGTAGAGCGCACCATTGGTAATGGTGAGGTCGGCA |
| Thr3_C27A_G43U_3prime | TGCTGATAGGCAGATTCGAACTGCCGACCTAACCCTT |
| Thr3_A28C_U42G_3prime | TGCTGATAGGCAGATTCGAACTGCCGACCTCCCCCTT |
| Thr3_C29U_G41A_3prime | TGCTGATAGGCAGATTCGAACTGCCGACCTCATCCTT |
| Thr3_C30G_G40C_3prime | TGCTGATAGGCAGATTCGAACTGCCGACCTCACGCTT |
| Thr3_C31A_G39U_3prime | TGGTGCTGATAGGCAGATTCGAACTGCCGACCTCACCATT |
| Thr3_G26A_body | GATATAGCTCAGTTGGTAGAGCACACCCTTGGTAAGGGTGAGGTCGGCA |
| Thr3_G34C_body | GATATAGCTCAGTTGGTAGAGCGCACCCTTCGTAAGGGTGAGGTCGGCA |
| Thr3_G34U_body | GATATAGCTCAGTTGGTAGAGCGCACCCTTTGTAAGGGTGAGGTCGGCA |
| Thr3_U3C_A70G_5prime | GCTAATACGACTCACTATAGCCGATATAGCTCAGTTGGTA |
| Thr3_U3C_A70G_3prime | TGGTGCCGATAGGCAGATTCGAACTGCCGACCTCACCCTT |
| Thr3_U62C_3prime | TGGTGCTGATAGGCGGATTCGAACTGCCGACCTCACCCTT |
| Thr3_A52G_U62C_body | GATATAGCTCAGTTGGTAGAGCGCACCCTTGGTAAGGGTGAGGTCGGCG |
| Thr3_A52G_U62C_3prime | TGGTGCTGATAGGCGGATTCGAACCGCCGACCTCACCCTT |
| Thr3_G35A_body | GATATAGCTCAGTTGGTAGAGCGCACCCTTGATAAGGGTGAGGTCGGCA |
| Thr3_G35C_body | GATATAGCTCAGTTGGTAGAGCGCACCCTTGCTAAGGGTGAGGTCGGCA |
| Thr4_5prime | GCTAATACGACTCACTATAGCCGACTTAGCT |
| Thr4_body | GCCGACTTAGCTCAGTAGGTAGAGCAACTGACTTGTAATCAGTAGGTCACCAGTTCGATTCC |
| Thr4_3prime | TGGTGCCGACTACCGGAATCGAACTGGTG |
| Thr4_ASL_1_body | GCCGACTTAGCTCAGTAGGTAGAGCGCACCCTTGGTAAGGGTGAGGTCACCAGTTCGATTCC |
| Thr4_U34G_body | GCCGACTTAGCTCAGTAGGTAGAGCAACTGACTTGTAATCAGTAGGTCACCAGTTCGATTCC |
| Thr4_A31C_U39G_body | GCCGACTTAGCTCAGTAGGTAGAGCAACTGCCTTGTAAGCAGTAGGTCACCAGTTCGATTCC |
| Thr4_A31C_U39G_U34G_body | GCCGACTTAGCTCAGTAGGTAGAGCAACTGCCTGGTAAGCAGTAGGTCACCAGTTCGATTCC |
| Human_Ser_5prime | GCTAATACGACTCACTATAGACGAGGTGGCCGAGTGGT |
| Human_Ser_body | GGTGGCCGAGTGGTTAAGGCGATGGACTGCTAATCCATTGTGCTTTGCAC |
| Human_Ser_3prime | TGGCGACGAGGATGGGATTCGAACCCACGCGTGCAAAGCACAATGGATTA |
| **Primers for cloning and site-directed mutagenesis** | |
| yaeB_Cm_f | aggctgtatgtatcggttctgcgcttaacattacggcgaaACCAGCAATAGACATAAGCGGC |
| yaeB_Cm_r | taccagtgcaaaagaaatgtcaaaagagaagggcgtgaatCTAAATCAGTAAGTTGGCAGC |
| hTrmO_1stPCR_f | GGCACCTCAGCAACCAGTAG |
| hTrmO_1stPCR_r | TGAACAGCCCAAATCAATCA |
| hTrmO_2ndPCR_f | AGTCAGTCgctagcATGCGCGGCTTGGAGG |
| hTrmO_2ndPCR_r | AGTCAGTCgtcgacTTAAGACCCTAGAGACACCAAGG |
| TrmO_pHSG415r_EcoRI_f | agctagctGAATTCccaatgctgtattactgcatagc |
| TrmO_pHSG415r_XhoI_r | agctagctCTCGAGagttggaaggtagaacaggc |
| Q25A_f | ccgttccgcgcGCgccaggtctggtaaaaa |
| Q25A_r | accagacctggcGCgcgcggaacggcgaact |
| R92A_f | ttttcgcaacaGCctctactttccgcccta |
| R92A_r | ggaaagtagagGCtgttgcgaaaaccccca |
| D125A_f | tcggcagtctggCtctggtcgatggtacgcc |
| D125A_r | ccatcgaccagaGccagactgccgagcttca |
| K136A_f | tagtggatatcGCaccgtatctcccctttg |
| K136A_r | ggagatacggtGCgatatccactaccggcg |
| D194A_R196A_f | actggcgcaggCcccgGCcccggcctatc |
| D194A_R196A_r | ccgggcgcgggGcctgcgccagtacttcg |
| Thr_f | tcgtcttcaagaattCgtgagtaaattaaaattttattgacttag |
| Thr_r | ATGTTTTTGGCGTCTTCCATggttgttacctcgttacctt |
| Fluc_f | ATGGAAGACGCCAAAAACA |
| Fluc_r | CCGGCGTAGAGGATCaaaataaacaaaagagtttgtagaaac |
| Del_thrL_f | aatataggcacgaccaaaggtaacgaggta |
| Del_thrL_r | cctttggtcgtgcctatattggttaaagta |
